# S-propargyl-cysteine attenuates temporomandibular joint osteoarthritis by regulating macrophage polarization via Inhibition of JAK/STAT signaling

**DOI:** 10.1186/s10020-025-01186-6

**Published:** 2025-04-07

**Authors:** Wenyi Cai, Antong Wu, Zhongxiao Lin, Wei Cao, Janak L. Pathak, Richard T. Jaspers, Rui Li, Xin Li, Kaihan Zheng, Yufu Lin, Na Zhou, Xin Zhang, Yizhun Zhu, Qingbin Zhang

**Affiliations:** 1 Department of Temporomandibular Joint, School and Hospital of Stomatology, Guangdong Engineering Research Center of Oral Restoration and Reconstruction, Guangzhou Key Laboratory of Basic and Applied Research of Oral Regenerative Medicine, Guangzhou Medical University, 195 Dongfeng Road (West), Yuexiu District, Guangzhou, Guangdong 510140 China; 2 Laboratory for Myology, Department of Human Movement Sciences, Faculty of Behavioural and Movement Sciences, Vrije Universiteit Amsterdam, Amsterdam Movement Science, Amsterdam, The Netherlands; 3 School of Pharmacy, State Key Laboratory of Quality Research in Chinese Medicines and Laboratory of Drug Discovery from Natural Resources and Industrialization, Macau University of Science and Technology, Room 210, Block E, Avenida Wai Long, Taipa, Macau China; 4 Guangzhou Municipal and Guangdong Provincial Key Laboratory of Molecular Target & Clinical Pharmacology, The NMPA and State Key Laboratory of Respiratory Disease, School of Pharmaceutical Sciences, Guangzhou Medical University, Guangzhou, 511436 China

**Keywords:** Temporomandibular joint osteoarthritis, S-propargyl-cysteine, Macrophage, JAK/STAT, Synovial, Hydrogen sulfide

## Abstract

**Background:**

Temporomandibular joint osteoarthritis (TMJ-OA) is a disease characterized by cartilage degradation and synovial inflammation, with limited effective treatment currently. Synovial macrophage polarization is pivotal in TMJ-OA progression, making it a promising therapeutic aspect. This study investigated the effects of S-propargyl-cysteine (SPRC), an endogenous H2S donor, on macrophage polarization and its therapeutic potential in alleviating TMJ-OA.

**Methods:**

A MIA-induced TMJ-OA rat model and LPS-stimulated RAW264.7 macrophages were employed to evaluate the effects of SPRC in vivo and in vitro. TMJ bone and cartilage were analyzed via micro-CT and histological methods, while macrophage polarization markers expression were assessed via RT-qPCR, western blot, and immunofluorescence. RNA sequencing was performed on macrophages, and the JAK2/STAT3 signaling pathway was validated using the JAK2-specific inhibitor AG490. The direct effects of SPRC on rat primary condylar chondrocytes were examined by evaluating ECM synthesis and degradation. Co-culture experiments further assessed macrophage-chondrocyte interactions.

**Results:**

SPRC significantly alleviated cartilage and bone damage in the TMJ-OA rat model, as demonstrated by improved bone volume and cartilage structure. SPRC reduced pro-inflammatory M1 macrophage infiltration and enhanced anti-inflammatory M2 macrophage polarization. SPRC effectively inhibited the JAK2/STAT3, leading to reduction of inflammatory markers, including TNF-α, IL-6, and iNOS. Co-culture experiments revealed that SPRC-treated macrophage-conditioned medium improved chondrocyte metabolic activity and restored ECM integrity.

**Conclusions:**

SPRC-modulated macrophage polarization alleviates TMJ-OA via JAK/STAT downregulation, thereby reducing synovial inflammation and cartilage degradation. These findings position SPRC as a promising therapeutic candidate for TMJ-OA and provide insights into novel strategies targeting macrophage polarization and synovium-cartilage crosstalk.

**Graphical abstract:**

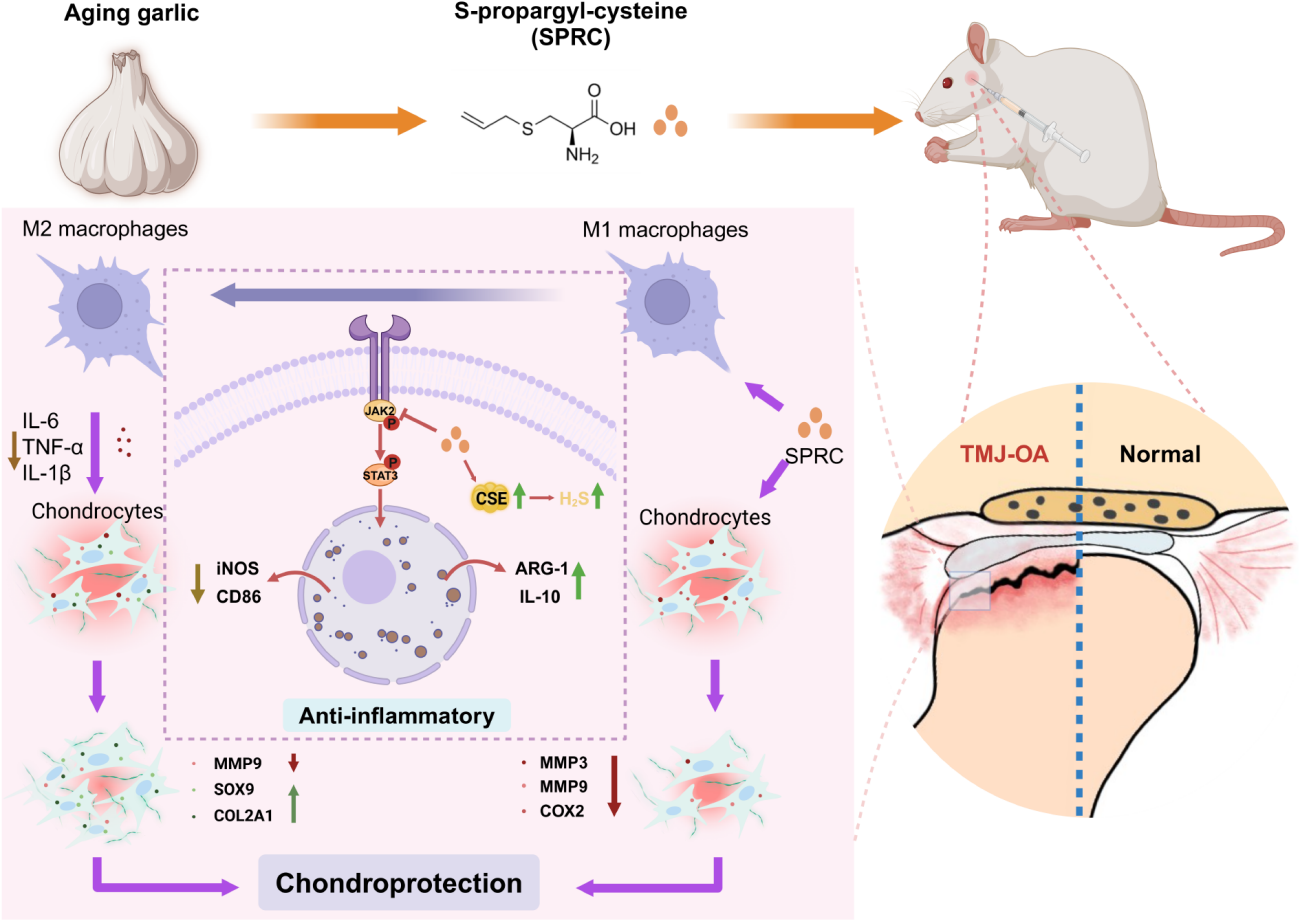

**Supplementary Information:**

The online version contains supplementary material available at 10.1186/s10020-025-01186-6.

## Background

Temporomandibular disorders (TMD) is a common oral disease with a prevalence of 34%, with a 9–56% higher prevalence in women than in men (Zieliński et al. 2024). Temporomandibular joint (TMJ) osteoarthritis (TMJ-OA), as an important subtype of TMD, is a low-inflammatory degenerative TMJ disease characterized by synovial inflammation and an imbalance between chondrocyte synthesis and catabolism, both of which are critical factors in its pathological progression (Cardoneanu et al. 2022). Females are more susceptible to TMJ-OA and tend to experience more severe symptoms (Lora et al. 2016). Current treatments of TMJ-OA primarily aim to manage symptoms by alleviating pain and restoring joint function. However, no effective therapies exist to regenerate cartilage or reduce synovial inflammation in patients with TMJ-OA (Wang et al. 2015).

The TMJ is composed of various cell types, including chondrocytes, synovial fibroblasts, synovial macrophages, osteoblasts, and osteoclasts (Li et al. 2021; Wu et al. 2024). Among these, synovial macrophages play a pivotal role in regulating TMJ-OA progression (Hu et al. 2024). In TMJ-OA, synovial fibroblasts release inflammatory cytokines, recruit synovial macrophages, and polarize them into the M1 pro-inflammatory phenotype (Ibi et al. 2018). M1 macrophages, in turn, produce pro-inflammatory cytokines, reactive oxygen species (ROS), and nitric oxide (NO), which exacerbate cartilage degradation (Lopa et al. 2015; Haltmayer et al. 2019). Reports from the literature and our previous study showed that suppressing M1 macrophages and promoting M2 anti-inflammatory macrophages can alleviate TMJ-OA and OA (Wu et al. 2023; Luo et al. 2023; Zhao et al. 2024; Zhou et al. 2019). Thus, targeting macrophage polarization represents a promising therapeutic strategy for reducing harmful inflammatory stimuli affecting chondrocytes by shifting M1 macrophages to an anti-inflammatory M2 phenotype (Xia et al. 2024).

Hydrogen sulfide (H₂S), the third identified endogenous gas transmitter after nitric oxide and carbon monoxide, exerts diverse effects on physiological and pathophysiological processes. H₂S is enzymatically synthesized from sulfur-containing amino acids, such as L-cysteine, by cystathionine β-synthase (CBS), cystathionine γ-lyase (CSE), and 3-mercaptopyruvate sulfurtransferase (3-MST) (Cirino et al. 2023; Wang 2002). H₂S exhibits anti-inflammatory, antioxidant, and cytoprotective properties (Bechelli et al. 2023). Importantly, H₂S is an endogenous regulator of the immune system (Dilek et al. 2020) that is capable of shifting macrophages from the M1 phenotype to the M2 phenotype and thereby mitigating inflammation (Zhou et al. 2023; He et al. 2024; Kumar et al. 2021). Recent studies have also shown that H₂S can reduce cartilage damage, underscoring its therapeutic potential for OA (Song et al. 2024; Wang et al. 2021a, b; Ha et al. 2015; Ma et al. 2024). However, the therapeutic efficacy of H_2_S in TMJ-OA treatment has not yet been investigated.

S-propargyl-cysteine (SPRC), a H₂S donor derived from aged garlic, promotes CSE expression and exhibits anti-inflammatory and antioxidant properties (Pan et al. 2011). Compared with other H_2_S donors, such as Fast-releasing donors NaHS or Natural donors DATS, SPRC demonstrates excellent biological safety, good stability, and a long half-life, allowing for sustained H₂S release (Hao et al. 2021; Wen and Zhu 2015; Tran et al. 2015). SPRC has demonstrated therapeutic potential in various conditions, including cardiovascular diseases (Ping et al. 2023; Tran et al. 2019), neurodegenerative disorders (Xi et al. 2023; Gong et al. 2011), and inflammatory diseases (Sidhapuriwala et al. 2012; Peng et al. 2021; Ma et al. 2022), such as Rheumatoid arthritis (RA). RA is an autoimmune disorder characterized by synovitis, with macrophages playing a critical role as key immune components in the synovial tissue (Jang et al. 2022). Notably, SPRC has been shown to mitigate the inflammatory response in RA. Existing literature suggests that SPRC reduces the expression of inflammatory mediators in IL-1β-induced human rheumatoid fibroblast-like synoviocytes (MH7A) in a concentration-dependent manner. Additionally, SPRC inhibits IL-1β-mediated MH7A cell migration and invasion. In vivo studies further demonstrate that SPRC mitigates inflammatory responses by modulating the Nrf2-ARE signaling pathway, alleviating adjuvant-induced arthritis in rats (Wu et al. 2016; Wang et al. 2021a, b). These findings highlight SPRC’s potential as a therapeutic agent for inflammatory diseases However, the role of SPRC in macrophage polarization and its potential to mitigate TMJ-OA progression remain unexplored. This study aimed to investigate the effects of SPRC on macrophage polarization and to evaluate its protective impact on condylar chondrocytes under inflammatory conditions. We developed a rat monoiodoacetate (MIA)-induced TMJ-OA model to assess the in vivo therapeutic potential of SPRC. The potential mechanisms underlying SPRC-induced macrophage polarization were also examined in vivo and in vitro. We found that SPRC-modulated macrophage polarization alleviates TMJ-OA by inhibiting the JAK/STAT signaling pathway, thereby reducing synovial inflammation and cartilage degradation.

## Methods

### Animal study

Twenty-four 8-week-old female Sprague–Dawley (SD) rats were procured from the Guangdong Medical Laboratory Animal Center. The rats were housed under specific pathogen-free conditions with access to standard food and water. Following a 1-week acclimatization period, the rats were randomly divided into four groups (*n* = 6 per group): Shame, MIA + normal saline (NS), MIA + SPRC, and MIA + hyaluronic acid (HA). The TMJ-OA model was established as described in our previous study (Hei et al. 2024), briefly, 0.5 mg MIA was injected into the bilateral TMJ cavity via an insulin needle. The Shame group underwent sham procedures without MIA injection. Two weeks after model induction, intra-articular injections were administered once weekly for 4 weeks. The MIA + NS group received 50 µL of NS, the MIA + SPRC group received 50 µL of SPRC (100 µM), and the MIA + HA group was treated with 50 µL of HA. All procedures complied with ethical standards and were approved by the Ethics Committee of Guangdong HUA WEI Testing Co., Ltd. (Approval No. 202310002).

### Pain behavioral measurement

The head withdrawal threshold (HWT) was assessed weekly to evaluate mechanical hyper-nociception in the TMJ region (Zhang et al. 2019). Measurements were performed via Von Frey filaments (IITC Life Science, USA), which apply calibrated mechanical stimuli to the preauricular area of each rat as described in our previous study (Hei et al. 2024). A single investigator conducted all the assessments under standardized environmental conditions to ensure consistency and reliability.

### Micro-CT scanning evaluation

TMJ tissue samples were harvested and fixed in 4% paraformaldehyde (PFA) for subsequent analysis. The samples were scanned coronally via a micro-CT system (Bruker, China) at 85 kV and 200 µA with an adequate pixel size of 10 μm. Key bone structure parameters, including the bone volume-to-total tissue volume ratio (BV/TV), trabecular separation (Tb.Sp), and bone mineral density (BMD), were quantified for evaluation.

### Histological observation

TMJ tissue samples were decalcified in 10% EDTA for 6 weeks, followed by dehydration, paraffin embedding, and sagittal sectioning at a thickness of 4 μm. The sections were stained with hematoxylin and eosin (H&E, Solarbio, China) and safranin-O/fast green (S&F, Solarbio, China). Articular cartilage degradation and synovial inflammation were evaluated via the Mankin OA scoring system and by assessing the synovial lining layer (Liu et al. 2023).

### Immunohistochemistry

Immunohistochemical analysis was performed using the following primary antibodies: anti-MMP13 (Proteintech, China), anti-COL2A1 (Arigobio, China), anti-iNOS (Signalway Antibody, USA), and anti-ARG-1 (Proteintech, China). The tissue sections were deparaffinized, rehydrated, and subjected to antigen retrieval in sodium citrate buffer (pH 6.0) via a microwave for 20 min. After being blocked with 5% bovine serum albumin (Solarbio, China) for 1 h, the sections were incubated overnight at 4 °C with primary antibodies. The next day, the sections were treated with horseradish peroxidase (HRP)-conjugated secondary antibody for 1 h at room temperature. Color development was performed via diaminobenzidine (Beyotime, China), with positive staining appearing as a brownish-yellow signal. Images were captured via a light microscope (Leica, Germany) for analysis.

### Macrophage culture and treatment

RAW264.7 murine macrophages were cultured in Dulbecco’s modified Eagle’s medium (DMEM) supplemented with 10% fetal bovine serum (FBS; ExCell, China) and 1% penicillin-streptomycin (Gibco, USA) at 37 °C in a humidified incubator with 5% CO₂. The in vitro experiments consisted of the control, lipopolysaccharide (LPS), and LPS + SPRC groups. Referring to our previous study, where 100 ng/mL of LPS was used to induce M1 macrophage polarization. As for the concentration of SPRC (100 µM), we based our selection on relevant literature (Wu et al. 2023; Ping et al. 2023; Li et al. 2023). In the LPS group, RAW264.7 macrophages were stimulated with 100 ng/mL *E. coli* LPS (Sigama‒Aldrich, USA) for 24 h to induce the M1 pro-inflammatory phenotype. For the LPS + SPRC group, macrophages were pretreated with 100 µM SPRC for 1 h before LPS stimulation for 24 h. The control group was maintained under standard culture conditions without treatment. In the subsequent response experiments, two additional groups were introduced: the LPS + AG490 group and the LPS + SPRC + AG490 group. In the LPS + AG490 group, cells were pretreated with 50 µM AG490 (MedChemExpress, HY-12000, USA) for 1 h, followed by stimulation with LPS for 24 h. In the LPS + SPRC + AG490 group, cells were pretreated with 50 µM AG490 and 100 µM SPRC for 1 h and then stimulated with LPS for 24 h.

### Rat primary condylar chondrocytes (rPCCs) extraction and culture

Rat primary condylar chondrocytes (rPCCs) were isolated from 3-week-old female Sprague-Dawley (SD) rats obtained from the Guangdong Medical Laboratory Animal Center, following a previously established protocol (Yang et al. 2024a, b). Cells from the first to third passages were used in subsequent experiments. Condylar tissues were minced into approximately 1 mm³ fragments and digested with 0.2% collagenase II (Sigma‒Aldrich, USA) at 37 °C for 4–6 h. After digestion, the cells were collected via centrifugation, resuspended in DMEM/F12 medium supplemented with 10% fetal bovine serum (FBS) and 1% penicillin-streptomycin, and cultured under standard conditions. The identity of the rPCCs was confirmed through toluidine blue staining (Solarbio, China) and immunofluorescence analysis with an anti-COL2A1 antibody (1:100, Abcam, UK).

### Effect of SPRC on cell viability

The effects of SPRC on the viability of RAW264.7 macrophages and rPCCs were assessed via a Cell Counting Kit-8 (CCK8, Dojindo, Japan). RAW264.7 macrophages (8 × 10³ cells/well) and rPCCs (5 × 10³ cells/well) were seeded into 96-well plates and treated with SPRC at concentrations of 0, 25, 50, 100, and 200 µM for 24 h. Following incubation, 100 µL of 10% CCK-8 solution was added to each well, and the plates were incubated for 2 h. The absorbance was measured at 450 nm via a microplate reader (Thermo Fisher, USA) to evaluate cell viability.

### H_2_S detection

RAW264.7 macrophages were seeded in 6-well plates at a density of 4 × 10⁴ cells per well and treated according to the experimental group protocols. The supernatant was collected, and H₂S levels were quantified via a H₂S content determination kit (Geruisi, China) following the manufacturer’s instructions.

### ELISA

After RAW264.7 macrophages were treated as described, the culture medium was replaced with a fresh medium, and the cells were incubated for an additional 12 h. The supernatant was collected and centrifuged to remove cell debris. The concentrations of IL-6 and TNF-α in the supernatant were measured via mouse ELISA kits (Elabscience, China) according to the manufacturer’s protocols.

### Nitric oxide (NO) detection

Following the treatments, the supernatant of the RAW264.7 macrophages was collected, and NO levels were measured via an NO Content Determination Kit (Beyotime, China) according to the manufacturer’s guidelines.

### Western blot

RAW264.7 macrophages and rPCCs were lysed in RIPA lysis buffer (Beyotime, China) supplemented with 1% protease and phosphatase inhibitors. Protein concentrations were quantified via a BCA protein assay kit (Beyotime, China). Equal amounts of protein were subjected to SDS-PAGE and transferred onto 0.45 μm–0.22 μm polyvinylidene difluoride membranes (Millipore, USA). The membranes were blocked with QuickBlock™ Blocking Buffer (Beyotime, China) for 1 h and incubated overnight at 4 °C with primary antibodies. The following day, the membranes were incubated with HRP-conjugated secondary antibodies at 37 °C for 1 h. The protein bands were visualized via an Ultra-sensitive ECL Chemiluminescence Kit (Epizyme Biomedical Technology, China). Details of the primary antibodies used for western blotting are provided in Table 1. We analyzed the original blot images using ImageJ software (Fiji, version 2.14.0). For each protein, measurements began at the first lane, ensuring that the same frame size was applied consistently across all lanes for accurate comparison. To obtain quantitative data, we selected “Measure” from the “Analyze” menu, which opened a measurement window displaying the results sequentially. The relative intensity of each target band was normalized to the corresponding internal reference protein (e.g., GAPDH) to calculate the final quantitative values. These values were then used to generate bar graphs using GraphPad Prism software (version 9.0) for comparison across different experimental groups.

**Table 1:** Primary antibodies used for western blot

Antibody	Dilution	Manufacturer	Cat.No.
anti-MMP9	1:1000	Abcam, UK	ab76003
anti-MMP3	1:1000	Abcam, UK	ab52915
anti-COX2	1:1000	CST, USA	12282T
anti-SOX9	1:1000	CST, USA	82630
anti-COL2A1	1:1000	Abcam, UK	ab188570
anti-iNOS	1:1000	Abcam, UK	ab178945
anti-ARG-1	1:1000	Proteintech, China	16001-1-AP
anti-CD86	1:1000	Proteintech, China	13395-1-AP
anti-JAK2	1:1000	CST, USA	3230T
anti-p-JAK2	1:500	Abmart, China	T56570S
anti-STAT3	1:1000	CST, USA	9139T
anti-p-STAT3	1:1000	CST, USA	9145T

### RNA extraction and real-time quantitative PCR (RT-qPCR)

Total RNA was isolated from cultured cells via an RNA extraction kit (EZBioscience, China) following the manufacturer’s instructions. The extracted RNA was reverse-transcribed into complementary DNA (cDNA) via a one-step RT-PCR kit (Accurate Biology, China). Real-time quantitative polymerase chain reaction (RT-qPCR) was performed using specific primers for the target genes. The primer sequences are listed in Table 2. The relative mRNA expression levels were calculated via the 2^−ΔΔCt method, with GAPDH used as the internal reference gene for normalization. 

**Table 2:** Primers used for RT-qPCR

Gene	Acc.No.	Primer sequence (5’-3’)	Product length (bp)
*Mus Gapdh*	NM_001411843.1	F: GGCATCGAAGGTGGAAGAGT R: AGGTGGTGAAGCAGGCATCT	113
*Mus Cd86*	NM_019388.3	F: ACGTATTGGAAGGAGATTACAGCT R: TCTGTCAGCGTTACTATCCCGC	147
*Mus Il-1β*	NM_008361.4	F: GAAATGCCACCTTTTGACAGTG R: TGGATGCTCTCATCAGGACAG	116
*Mus Tnf-α*	NM_013693.3	F: CCTGTAGCCCACGTCGTAG R: GGGAGTAGACAAGGTACAACCC	148
*Mus Il-10*	NM_010548.2	F: AGCCTTATCGGAAATGATCCAGT R: GGCCTTGTAGACACCTTGGT	229
*Mus Arg-1*	NM_007482.3	F: TGTCCCTAATGACAGCTCCTT R: GCATCCACCCAAATGACACAT	204
*Mus Il-4*	NM_021283.2	F: GGTCTCAACCCCCAGCTAGT R: GCCGATGATCTCTCTCAAGTGAT	102
*Mus Il-6*	NM_001314054.1	F: CTGCAAGAGACTTCCATCCAG R: AGTGGTATAGACAGGTCTGTTGG	131
*Mus inos*	NM 010927.4	F: CAGAAGTGCAAAGTCTCAGACAT R: GTCATCTTGTATTGTTGGGCT	150
*Rat Gapdh*	NM_017008.4	F: CCATCAACGACCCCTTCATT R: CACGACATACTCAGCACCAGC	194
*Rat Mmp3*	NM_133523.3	F: TGAAGATGACAGGGAAGC R: CTGGAGAATGTGAGTGGG	235
*Rat Mmp9*	NM_031055.2	F: CTTGAAGTCTCAGAAGGTGGATC R: CGCCAGAAGTATTTGTCATGG	135
*Rat Mmp13*	NM_133530.1	F: TGACCCAGCCCTATCCCT R: ACCCTCCATAATGTCATACCC	244
*Rat Sox9*	NM_080403.3	F: AAGAGGCCACCGAACAGACT R: GACCCTGAGATTGCCCGGAG	132
*Rat Col2a1*	NM_012929.2	F: GGCCAGGATGCCCGAAAAT R: GTCACCTCTGGGTCCTTGTTC	129
*Rat Aggrecan*	NM_022190.2	F: GACCTGTGTGAGATCGACCA R: GGTCGGGAAAGTGGCGATAA	82

### Immunofluorescence staining

The cells were fixed with 4% PFA for 15 min and permeabilized with 0.5% Triton X-100 for 5 min. To block nonspecific binding, the samples were incubated in 3% bovine serum albumin (Solarbio, China) for 30 min at room temperature. The samples were subsequently incubated overnight at 4 °C with primary antibodies, including anti-iNOS, anti-ARG-1, and anti-CD86. The following day, the samples were treated with fluorescently labeled secondary antibodies for 1 h at room temperature, followed by counterstaining with 4′,6-diamidino-2-phenylindole for 5 min. Images were acquired via a confocal fluorescence microscope (Leica Microsystems, Wetzlar, Germany) to visualize the immunofluorescent signals.

### Conditioned medium (CM) collection and stimulation

CM was collected from three groups of macrophage cultures: Ctrl-CM (from untreated macrophages), LPS-CM (from LPS-treated macrophages), and LPS + SPRC-CM (from LPS- and SPRC-treated macrophages). After the treatment, the original medium was replaced with a fresh serum-free medium, and the CM was collected after 12 h. The CM was centrifuged at 1000×g for 5 min and stored at − 80 °C. The CM was mixed 1:1 with DMEM/F12 containing 10% FBS and used to culture the rPCCs for 7 days. Chondrogenic differentiation ability was evaluated by RT-qPCR, western blot, and toluidine blue staining.

### Toluidine blue staining

rPCCs (3 × 10^4^ cells/well) were seeded in 48-well plates and cultured with Ctrl-CM, LPS-CM, or LPS + SPRC-CM for 7 days. The cells were then fixed with 4% PFA for 15 min and stained with toluidine blue (Solarbio, China) to evaluate chondrogenic differentiation. Images were captured via a light microscope (Leica Microsystems, Germany).

### Statistical analysis

All the data are presented as the means ± standard deviation (SD). Statistical analyses were performed via GraphPad Prism software (version 9.0). Group comparisons were conducted via one-way analysis of variance (ANOVA), followed by Tukey’s post hoc test for multiple comparisons. The Kruskal–Wallis test was employed for non-normally distributed data, followed by Dunn’s test for pairwise comparisons. A *p*-value of less than 0.05 was considered statistically significant.

## Results

### SPRC attenuated condylar bone and cartilage damage in vivo

**Fig. 1:**
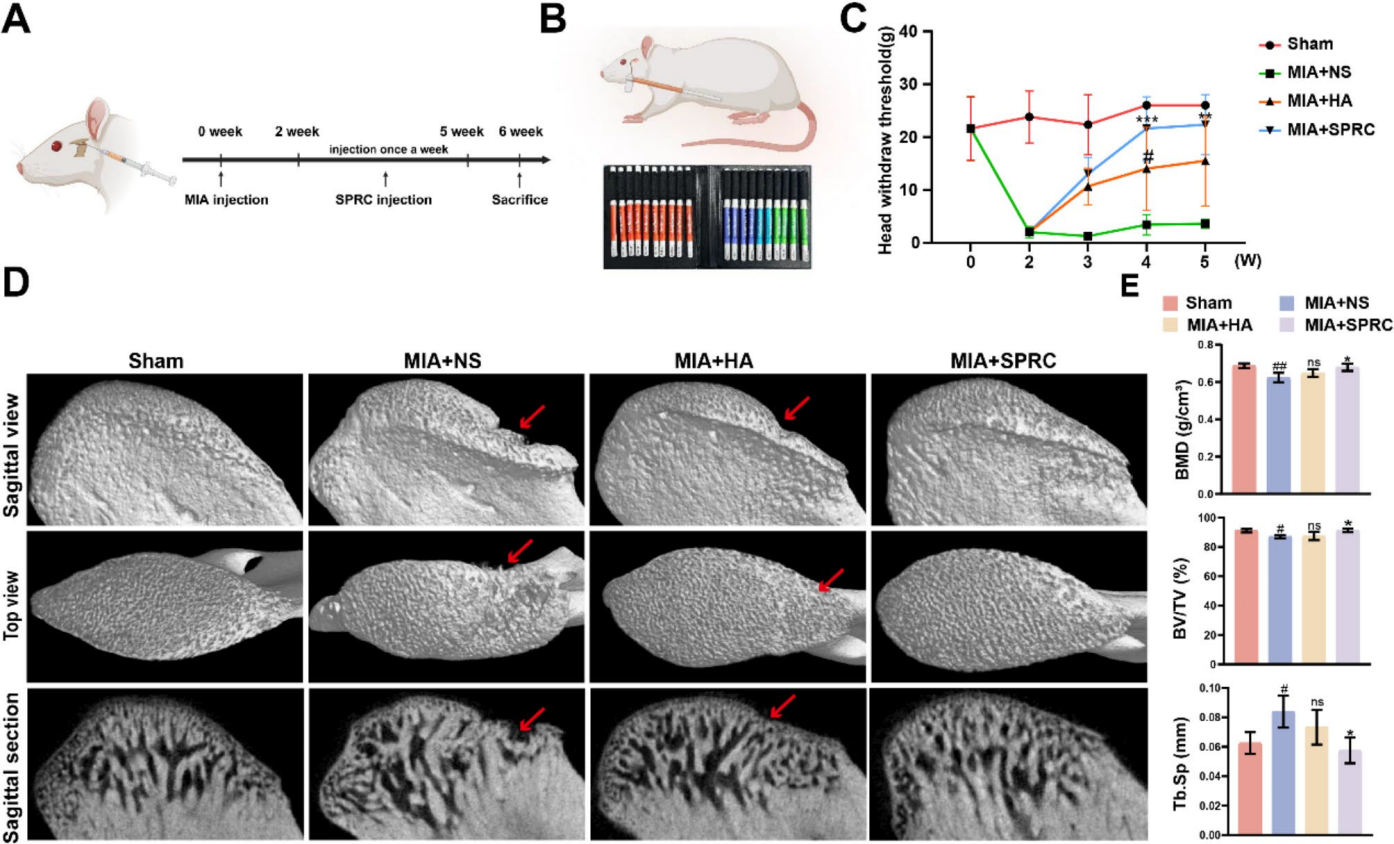
SPRC promoted subchondral bone remodeling and reduced pain in TMJ-OA rats. (**A**) Experimental timeline outlining TMJ-OA induction and SPRC treatment in vivo. (**B**) Schematic representation of pain behavior assessment and the time-dependent changes in pain behavior observed during the experiment, including baseline measurements before MIA induction and post-treatment evaluation with SPRC. (**C**) Representative sagittal views, top views, and sagittal sections of the condyles. (**D**) Quantitative analysis of subchondral bone parameters, including BMD, BV/TV, and Tb.Sp. The data were analyzed via one-way ANOVA (*n* = 3). Statistical significance is denoted as follows: ^#^*p* < 0.05 and ^##^*p* < 0.01 compared with the sham group; ^*^p < 0.05, ^**^p < 0.01, and ^***^p < 0.001 compared with the MIA + NS group. “ns” indicates no significant difference. Red arrows indicate bone defects

An MIA-induced TMJ-OA rat model was established to assess the therapeutic efficacy of SPRC in TMJ-OA in vivo. Distinct condylar morphological changes, cartilage defects, and pannus formation were observed 2 weeks post-MIA injection (Figure S1). HA represents a well-established intra-articular injection therapy, clinically proven to enhance joint lubrication, alleviate pain, and decelerate degenerative progression. Its therapeutic mechanisms are largely attributed to its role as a critical component of synovial fluid, which augments lubrication, reduces frictional forces, mitigates pain, and ultimately improves joint functionality. Therefore, HA was selected as the positive control in this study (Shao et al. 2023; Triantaffilidou et al. 2013). H&E staining results further confirmed the successful induction of the TMJ-OA model in vivo. The schematic diagram in Fig. 1A outlines the in vivo procedure for TMJ-OA induction and SPRC treatment. Figures 1B and C display the instruments and measurement methods used for pain behavior. Pain behavioral assessments revealed that rats with MIA-induced TMJ-OA presented increased sensitivity to mechanical stimulation. Notably, pain responses in the TMJ area were significantly reduced in the SPRC-treated group compared with the NS and HA groups. Micro-CT imaging revealed significant cartilage and subchondral bone damage following MIA injection, which was effectively mitigated by SPRC treatment. Compared with those in the control group, quantitative analysis of the subchondral bone parameters from the micro-CT data revealed a decrease in BMD and BV/TV, along with an increase in Tb. Sp, in the MIA + NS group compared to the control (Fig. 1D). SPRC treatment significantly ameliorated these bone alterations induced by MIA. The bone structural parameters (BV/TV, Tb.Sp, and BMD) in the SPRC treatment group returned to levels similar to those of the healthy control group. This indicates that SPRC has a strong ability to repair condylar bone.

**Fig. 2:**
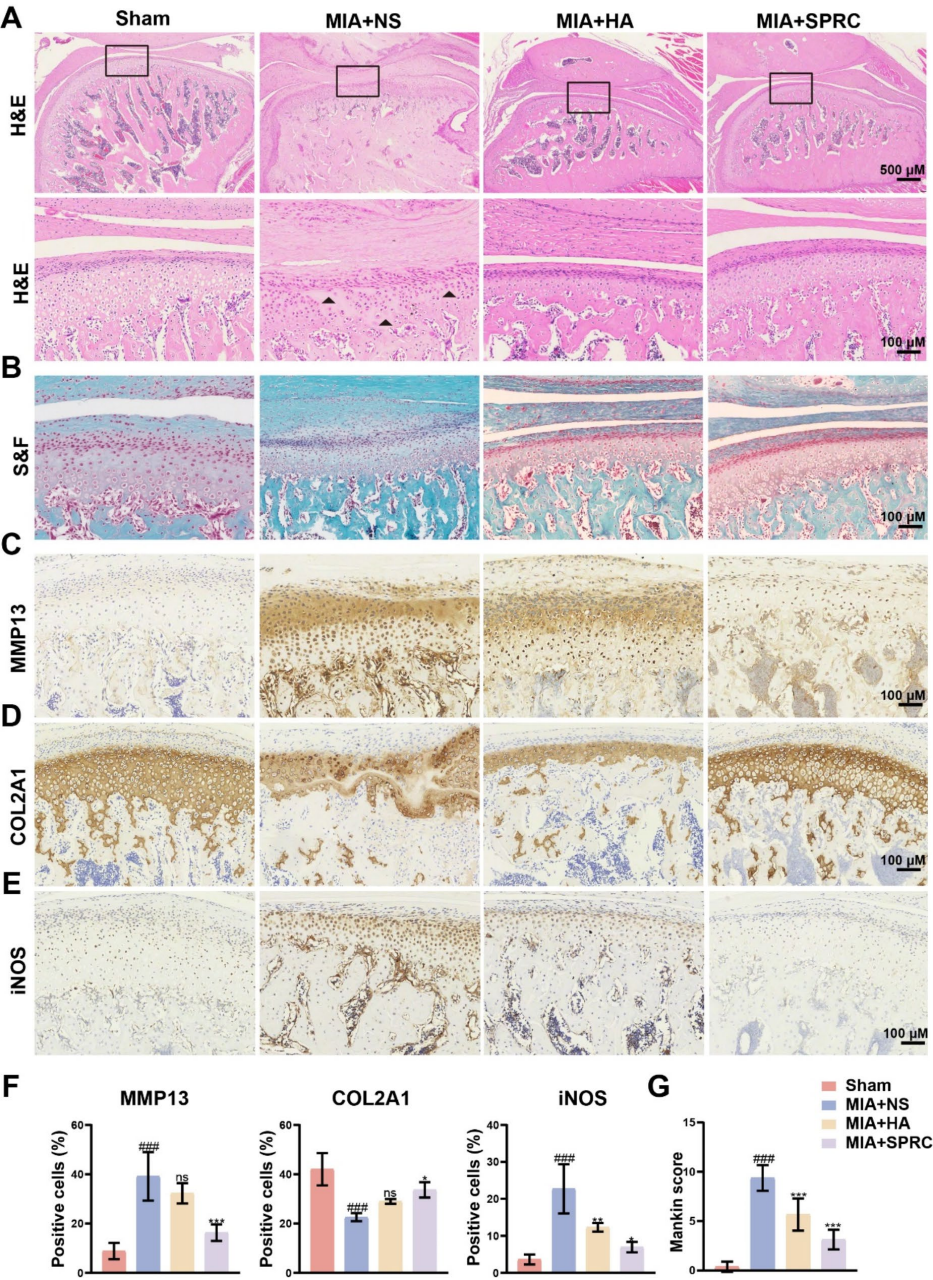
SPRC reduced condylar cartilage damage in TMJ-OA rats. (**A-B**) Representative images of condylar cartilage stained with H&E and S&F. (**C-E**) Representative images of immunohistochemical staining showing the expression of COL2A1, MMP13, and iNOS. (**F**) Quantitative analysis of COL2A1, MMP13, and iNOS expression levels. (**G**) Mankin score assessment of cartilage integrity. The data were analyzed via one-way ANOVA (*n* = 3). Statistical significance is denoted as follows: ^###^*p* < 0.001 compared with the sham group; ^*^p < 0.05, ^**^p < 0.01, and ^***^p < 0.001 compared with the MIA + NS group. “ns” indicates no significant difference. The black triangle indicates a decrease in the number of chondrocytes

Histological analysis with H&E and S&F staining revealed characteristic cartilage and subchondral bone destruction in the MIA + NS group. In contrast, the SPRC-treated group displayed significant restoration of TMJ anatomical structures, with preserved articular and calcified cartilage integrity, reduced subchondral bone degeneration, increased chondrocyte density, and enhanced proteoglycan staining in the condylar cartilage layer (Fig. 2A and B). Immunohistochemical analysis revealed that the expression of MMP13, a matrix metalloproteinase, was upregulated in the condylar cartilage of the MIA + NS group than in that of the control group but was lower in the SPRC-treated group. Similarly, iNOS was upregulated in the MIA + NS group but significantly downregulated following HA and SPRC treatment. Collagen II (COL2A1), a major cartilage component, was reduced in the condylar cartilage of the MIA + NS group compared with the control group (Fig. 2C and E), whereas COL2A1 levels were restored in the TMJ after SPRC treatment (Fig. 2D). Mankin scores for each group are shown in Fig. 2G, with a significant increase in the Mankin score observed in the TMJ-OA group. In contrast, compared with the MIA + NS group, the SPRC treatment group presented a significant decrease in the Mankin score. Furthermore, throughout the experiment, no adverse reactions were observed in rats, consistent with previous studies (Zheng et al. 2012). Figure S1D indicates that local SPRC injection does not induce systemic toxicity, as evidenced by the absence of histopathological changes in the heart, liver, spleen, lung, and kidney in the SPRC-treated group compared with those in the control group. These results indicate a favorable biosafety profile for SPRC.

### SPRC reduced synovial inflammation and the M1 phenotype of macrophages *in vivo*

**Fig. 3:**
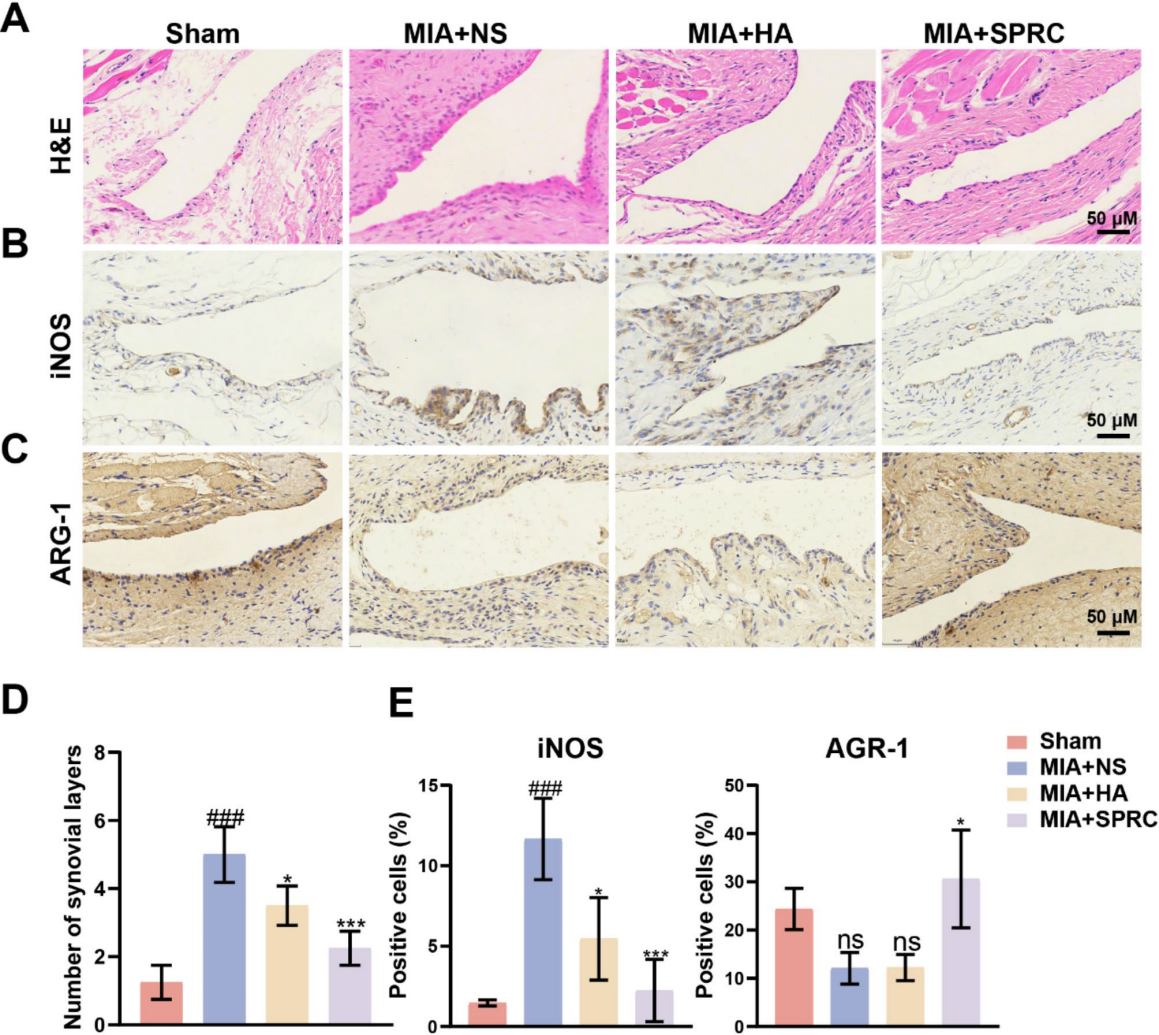
SPRC reduced synovial inflammation and M1-phenotype macrophages in TMJ-OA rats. (**A**) Representative images of synovial tissue stained with H&E. (**B-C**) Representative images of immunohistochemical staining showing the expression of iNOS and ARG-1. (**D**) Quantitative analysis of the number of synovial lining layers. (**E**) Quantitative analysis of iNOS and ARG-1 expression levels. The data were analyzed via one-way ANOVA (*n* = 3). Statistical significance is denoted as follows: ^###^*p* < 0.001 compared with the sham group; ^*^p < 0.05 and ^***^p < 0.001 compared with the MIA + NS group. “ns” indicates no significant difference

The synovial membrane has a two-layer structure consisting of an inner layer and a lower layer. The inner layer comprises 2 to 3 layers of macrophages and fibroblastic synovial cells, while the lower layer consists of fibrous connective tissue, blood vessels, and a few lymphocytes and macrophages. Proliferating macrophage infiltration in both layers is the primary feature of synovitis (Wu et al. 2024). As shown in Fig. 3A and D, the MIA + NS group exhibited significant thickening of the synovial layer and increased inflammatory cell infiltration. SPRC and HA treatment obviously reduced both synovial inflammation and synovial layer thickening. In the TMJ-OA synovium, the number of F4/80-positive (macrophage-labeled) cells increase significantly, as does the proportion of iNOS (M1 macrophage-labeled) and CD86-positive (M1 macrophage-labeled) cells (Xue et al. 2018). Compared with the MIA + NS group, the SPRC and HA groups presented significantly lower iNOS expression and inhibited M1 macrophage infiltration in the TMJ of MIA-induced TMJ-OA rats (Fig. 3B). Notably, SPRC treatment increased the expression of ARG-1, a marker of M2 macrophages with anti-inflammatory functions (Fig. 3C). These findings suggest that SPRC exerts anti-inflammatory effects by modulating macrophage polarization and reducing synovial inflammation.

### SPRC mediated the M1 to M2 macrophage phenotype polarization transition *in vitro*

**Fig. 4:**
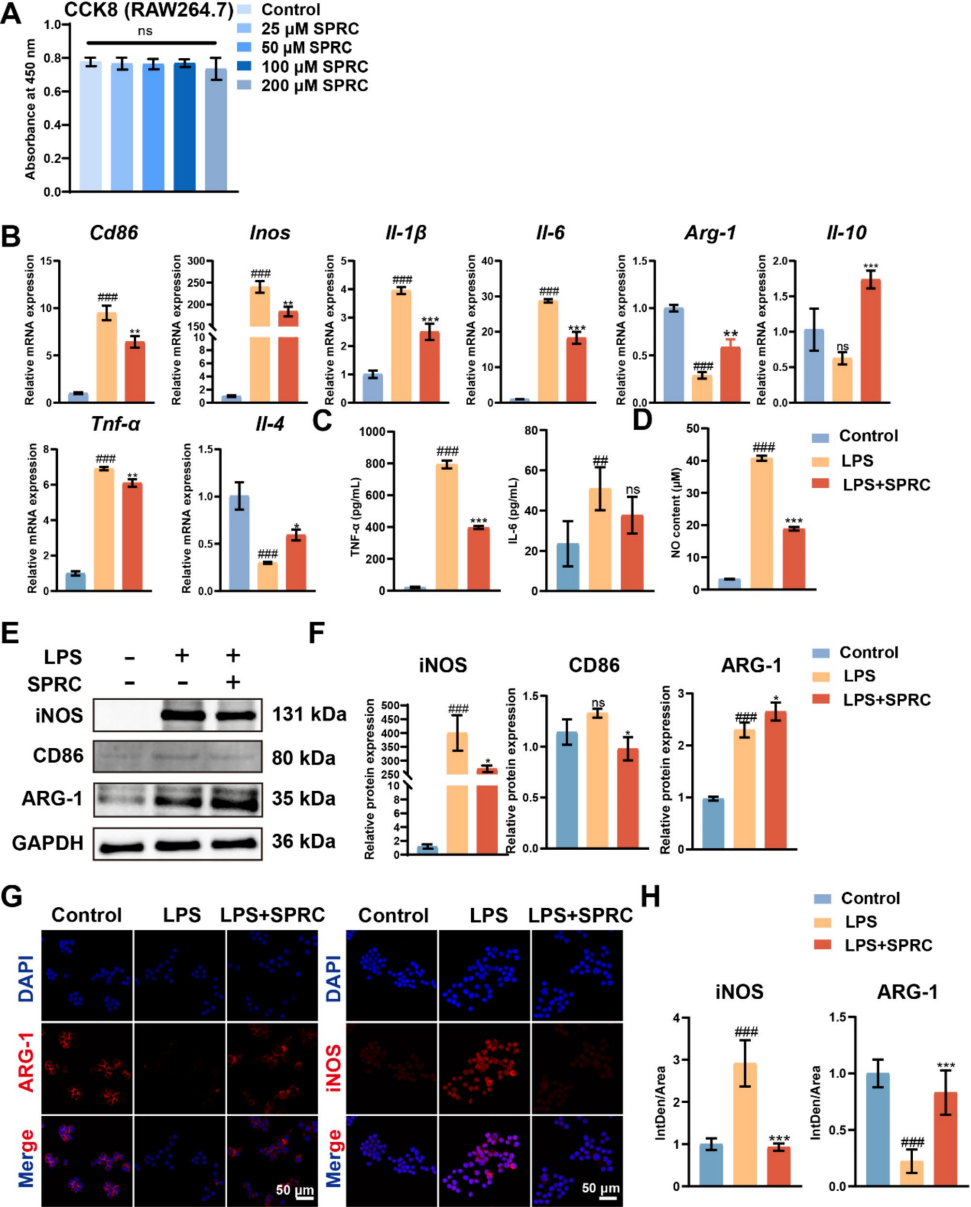
SPRC mediated the M1 to M2 macrophage phenotype polarization transition in vitro. (**A**) RAW264.7 macrophages were treated with various concentrations of SPRC (25, 50, 100, or 200 µM) for 24 h, and cell viability was evaluated via the CCK8 assay. (**B**) RT-qPCR analysis was conducted to measure the expression levels of M1-related genes (*Cd86*,* Inos*,* Il-6*,* Il-1β*, and *Tnf-α*) and M2-related genes (*Arg-1*,* Il-10*,* and Il-4*). (**C**) The levels of IL-6 and TNF-α in the supernatant of the culture medium were quantified via ELISA. (**D**) NO content in the culture medium supernatant was also assessed. (**E-F**) The protein levels of M1 macrophage markers (CD86 and iNOS) and the M2 macrophage marker ARG-1 were evaluated via western blot analysis. (**G-H**) Immunofluorescence staining for iNOS and ARG-1 was performed to assess macrophage polarization further. Statistical analysis was conducted via one-way ANOVA (*n* ≥ 3). Statistical significance is denoted as follows: ^#^*p* < 0.05 and ^###^*p* < 0.001 compared with the control group; ^*^p < 0.05, ^**^p < 0.01, and ^***^p < 0.001 compared with the LPS group. “ns” indicates no significant difference

To investigate the mechanism by which SPRC reduces synovial inflammation and the M1 macrophage phenotype, TMJ-OA synovitis was modeled in vitro using LPS-treated RAW264.7 macrophages. First, SPRC at concentrations of 20, 50, 100, and 200 µM did not affect the metabolic activity of RAW264.7 macrophages (Fig. 4A). Based on previous studies of SPRC, a concentration of 100 µM was selected for subsequent experiments (Ping et al. 2023; Ma et al. 2011). LPS treatment induced a strong M1-like pro-inflammatory phenotype, as evidenced by increased expression of CD86, iNOS, IL-1β, IL-6, and TNF-α and decreased expression of IL-4, IL-10, and ARG-1. SPRC treatment promoted a shift in macrophage polarization from the M1 to the M2 phenotype, as evidenced by decreased M1 marker expression and increased M2 marker expression at both the gene and protein levels, as confirmed by RT-qPCR, Western blot, and immunofluorescence analyses (Fig. 4B-H). Additionally, M1 macrophages produce TNF-α, IL-6, and NO, contributing to inflammatory responses (Shapouri-Moghaddam et al. 2018). SPRC reduced the LPS-induced increases in TNF-α, IL-6, and NO levels in macrophages, as confirmed by ELISA of the cell supernatant (Fig. 4C and D). These findings indicate that SPRC may mitigate inflammation by shifting macrophage polarization toward the anti-inflammatory M2 phenotype.

In mammals H_2_S is synthesized by CBS, CSE/CTH, and 3-MST, which catalyze the conversion of homocysteine to H_2_S (Cirino et al. 2023). SPRC, an endogenous H_2_S donor, can significantly increase CSE protein expression, promoting H2S production (Figure S2).

### SPRC treatment downregulated the JAK/STAT signaling pathway

**Fig. 5:**
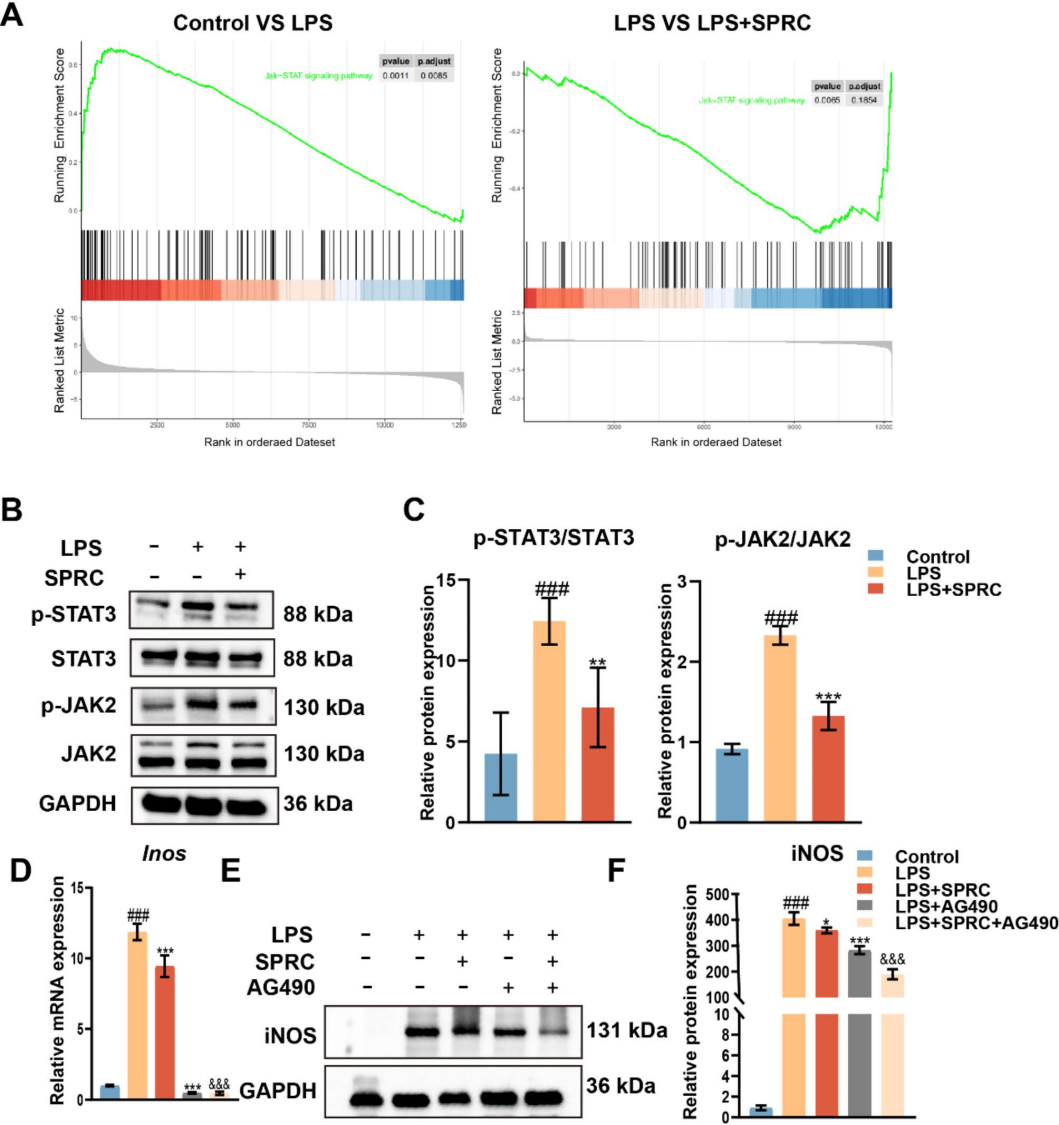
SPRC inhibited the JAK/STAT signaling pathway in vitro. (**A**) GSEA revealed significant enrichment of the JAK/STAT signaling pathway. (**B-C**) Western blot analysis of phosphorylated STAT3 (p-STAT3), total STAT3, phosphorylated JAK2 (p-JAK2), and total JAK2. (**D**) RT-qPCR analysis of Inos expression. (**E-F**) Western blot analysis of iNOS protein levels. The data were analyzed via one-way ANOVA (*n* ≥ 3). Statistical significance is denoted as follows: ^###^*p* < 0.001 compared with the control group; ^*^p < 0.05, ^**^p < 0.01, ^***^p < 0.001 compared with the LPS group; ^&&&^*p* < 0.001 compared with the LPS + AG490 group

RNA sequencing was conducted on RAW264.7 cells treated with LPS ± SPRC. As shown in Fig. 5A, Gene set enrichment analysis (GSEA) pathway enrichment revealed that the JAK/STAT signaling pathway was significantly enriched in the LPS-treated macrophages compared with the control macrophages. In contrast, these pathways were significantly downregulated in SPRC + LPS-treated cells compared with those treated with LPS alone. Western blot analysis further demonstrated that LPS treatment markedly increased the phosphorylation of JAK and STAT in macrophages. However, SPRC treatment significantly reduced the LPS-induced phosphorylation of both JAK and STAT (Fig. 5B and C). These findings are consistent with the results of the mRNA sequencing analysis. To further verify the effect of SPRC on the JAK/STAT signaling pathway, the JAK2 inhibitor AG490 was used. The results indicated that inhibition of the JAK/STAT pathway by AG490 reduced the expression of the M1 macrophage marker iNOS, mirroring the effect of SPRC. Moreover, this downregulation was accompanied by a reduction in the expression of pro-inflammatory cytokines, suggesting that SPRC exerts anti-inflammatory effects by inhibiting the JAK/STAT signaling pathway in macrophages (Fig. 5D-F).

### SPRC reduced condylar chondrocyte extracellular matrix (ECM) catabolism in vitro

**Fig. 6:**
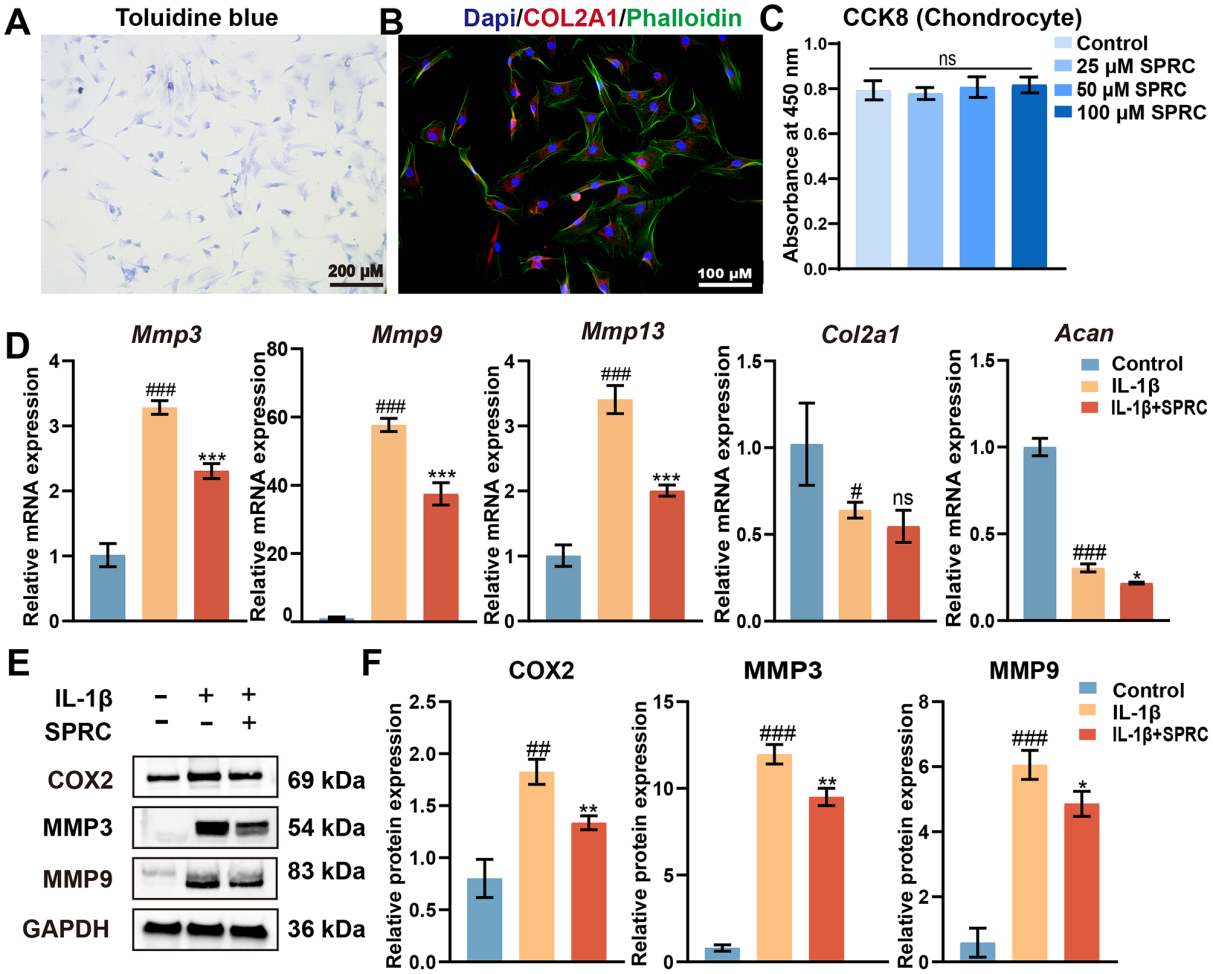
SPRC reduced condylar chondrocytes ECM catabolism in vitro. (**A**) Representative images of condylar chondrocytes stained with toluidine blue to assess extracellular matrix (ECM) integrity. (**B**) Immunofluorescence staining of chondrocytes showing DAPI-stained nuclei (blue), phalloidin-stained F-actin (green), and COL2A1 (red). (**C**) Cell viability of rat primary condylar chondrocytes (rPCCs) treated with SPRC (25, 50, or 100 µM) for 24 h, as evaluated by the CCK8 assay. (**D**) RT-qPCR analysis of matrix degradation-related genes (Mmp3, Mmp9, and Mmp13) and matrix synthesis-related genes (Col2a1 and Acan). (**E-F**) Western blot analysis of COX2, MMP3, and MMP9 protein expression levels. The data were analyzed via one-way ANOVA (*n* ≥ 3). Statistical significance is denoted as follows: ^#^*p* < 0.05, ^##^*p* < 0.01, and ^###^*p* < 0.001 compared with the control group; ^*^p < 0.05, ^**^p < 0.01, and ***p < 0.001 compared with the IL-1β group. “ns” indicates no significance

Chondrocytes can synthesize and secrete ECM, which plays a critical role in maintaining cartilage tissue’s load-bearing and cushioning functions on joint surfaces (Li et al. 2021). As an essential component of the condylar articular surface, it is crucial to investigate the direct effects of SPRC on chondrocytes. As shown in Fig. 6A and B, rPCCs were identified via toluidine blue and COL2A1-red staining. A CCK-8 assay was used to evaluate the cytotoxic effects of SPRC on rPCCs. The results demonstrated that SPRC concentrations of 20, 50, and 100 µM did not influence the metabolic activity of rPCCs (Fig. 6C). Consequently, the subsequent experiments were conducted at a concentration of 100 µM, consistent with prior conditions. In vitro models of TMJ-OA are commonly established via the induction of cytokines, such as IL-1β (Chen et al. 2024). Under IL-1β stimulation, rPCCs presented elevated expression of MMPs, including MMP3, MMP9, and MMP13, but reduced expression of chondrocyte-related genes such as Col2a1 and Aggrecan. SPRC treatment significantly mitigated ECM degradation in IL-1β-induced condylar chondrocytes, as evidenced by the downregulation of MMPs but not the upregulation of COL2A1 and aggrecan levels. (Figs. 6D-F). Cyclooxygenase 2 (COX2) is an inducible enzyme that is minimally expressed under normal conditions but is upregulated in response to inflammatory stimuli. The IL-1β group presented significant COX2 overexpression, whereas SPRC treatment reduced COX2 levels (Fig. 6E **and F**). These findings suggest that SPRC protects rPCCs from inflammatory damage and ECM degradation, suggesting potential therapeutic benefits for TMJ-OA treatment.

### SPRC-treated macrophages restored metabolic activity, chondrogenic differentiation and matrix destruction in rPCCs

**Fig. 7:**
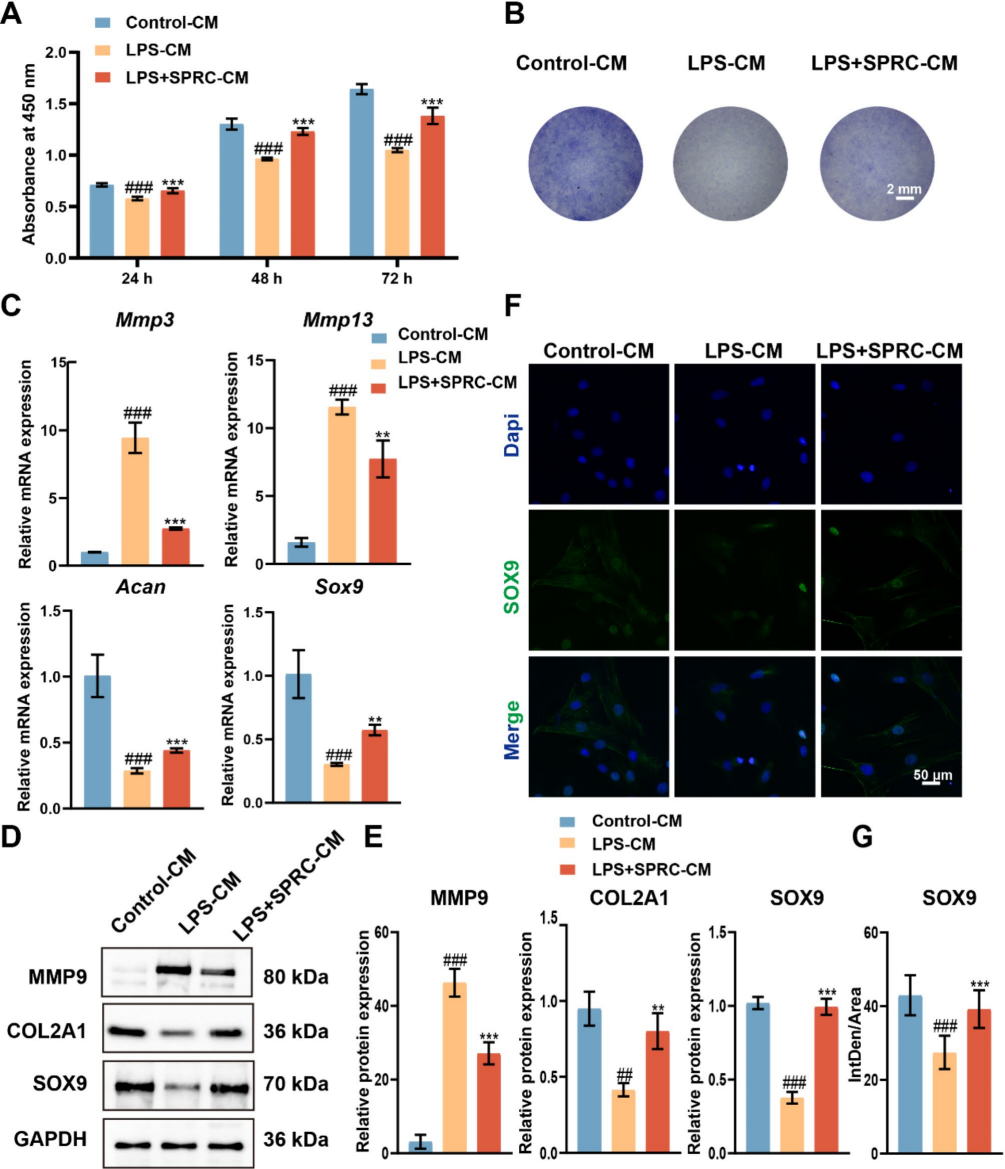
CM from SPRC-treated macrophages restored metabolic activity, chondrogenic differentiation, and matrix destruction in rPCCs. (**A**) The metabolic activity of rPCCs cultured with CM from macrophages was evaluated at 24, 48, and 72 h via the CCK8 assay. (**B**) Representative images of toluidine blue-stained rPCCs cultured with macrophage CM for 7 days to assess ECM integrity. (**C**) RT-qPCR analysis of matrix degradation-related genes (Mmp3, Mmp13) and matrix synthesis-related genes (Acan, Sox9) in rPCCs. (**D-E**) Western blot analysis of MMP9, COL2A1, and SOX9 protein expression levels in rPCCs cultured with macrophage CM. (**F-G**) Immunofluorescence staining of SOX9 in rPCCs to assess chondrogenic differentiation. The data were analyzed via one-way ANOVA (*n* ≥ 3). Statistical significance is denoted as follows: ^##^*p* < 0.01, ^###^*p* < 0.001 compared with the control-CM group; **p < 0.01, ^***^p < 0.001 compared with the LPS-CM group

Co-culturing macrophages and chondrocytes allows for a better understanding of their interactions and the underlying mechanisms of inflammation and repair (Wu et al. 2023; Jin et al. 2022). An in vitro model of TMJ-OA can be effectively established by treating chondrocytes with LPS-induced M1-polarized macrophage-CM. Figure 7A-E shows that compared with control CM, CM derived from LPS-treated macrophages significantly inhibited cell viability and ECM synthesis in rPCCs while promoting ECM degradation. Specifically, the CCK-8 assay revealed reduced cell activity, and RT-qPCR and Western blot analysis showed increased expression of MMP3, MMP9, and MMP13 and decreased expression of COL2A1, ACAN, and SOX9. Additionally, toluidine blue staining showed a significant reduction in both the area and density of the ECM. However, CM from SPRC-treated macrophages restored metabolic activity, chondrogenic differentiation, and matrix integrity in rPCCs. Immunofluorescence analysis also revealed increased SOX9 expression in the LPS + SPRC-CM group compared with the LPS-CM group (Fig. 7F and G). These findings provide deeper insight into how SPRC modulates TMJ-OA and supports cartilage health under these conditions.

## Discussion

TMJ-OA is a multifactorial disease characterized by cartilage degradation, bone destruction, synovitis, and joint pain (Wang et al. 2023; Krisjane et al. 2012). Synovial macrophages, vital immune cells within synovial tissue, are critical in maintaining joint homeostasis by regulating synovial fluid production, clearing cellular debris, and eliminating pathogens (Kurowska-Stolarska and Alivernini 2022). Synovial inflammation is a significant driver of TMJ-OA progression, with pro-inflammatory M1 macrophages contributing to synovitis by releasing inflammatory mediators and MMPs, which exacerbate cartilage and bone degeneration (Zhang et al. 2020). Consequently, reducing synovial inflammation and promoting cartilage regeneration are vital therapeutic strategies for treating TMJ-OA. This study demonstrated the therapeutic potential of SPRC in treating TMJ-OA through the modulation of macrophage polarization. Specifically, SPRC decreased M1 macrophage polarization and increased M2 macrophage polarization both in vivo and *in vitro.* Furthermore, SPRC significantly inhibited the secretion of pro-inflammatory cytokines, including IL-6, TNF-α, and iNOS; downregulated the expression of M1 macrophage markers (CD86 and iNOS); and upregulated the expression of M2 macrophage markers such as ARG-1 (See Graphical Abstract).

Studies have demonstrated that activation of the JAK/STAT pathway contributes to the pathological progression of OA by regulating inflammation (Kaneko et al. 2000), immune responses (Yang et al. 2024a, b), mechanical load (Millward-Sadler et al. 2006), and apoptosis (Yang et al. 2016), ultimately leading to cartilage destruction, subchondral bone remodeling, and synovial inflammation (Zhou et al. 2022; Li et al. 2017). In this study, mRNA sequencing coupled with GSEA revealed significant enrichment of the JAK/STAT signaling pathway in macrophages stimulated with LPS compared with the control group. Notably, SPRC treatment markedly downregulated this pathway. Cellular experiments further confirmed that LPS stimulation increased the phosphorylation of JAK2 and STAT3, critical components of the JAK/STAT pathway. However, SPRC treatment significantly attenuated the phosphorylation levels, demonstrating efficacy comparable to the JAK2-specific inhibitor AG490. These findings underscore the potential of SPRC to inhibit JAK2/STAT3 pathway activation. In addition to the JAK/STAT signaling pathway, our RNA-seq data analysis identified several other pathways that influence SPRC-mediated macrophage polarization, including TNF and PI3K/Akt. These pathways play crucial roles in the transition of pro-inflammatory M1 macrophages to tissue-repairing M2 macrophages (Zheng et al. 2024; Vergadi et al. 2017; Sawoo et al. 2021).

SPRC functions as an endogenous donor of H₂S by upregulating the expression of CSE, a key enzyme in H₂S biosynthesis (Wang et al. 2009). H₂S has been widely recognized as a mediator in inflammatory joint diseases and has been shown to exhibit anti-inflammatory, anti-catabolic, and antioxidant properties in vitro models of synovial cells and articular chondrocytes derived from RA and OA tissues (Burguera et al. 2017; Geng et al. 2023). Consistent with these findings, previous studies have shown that CSE-mediated H₂S production alleviates inflammation and pain in OA (Wei et al. 2023). In this study, SPRC was found to increase H₂S levels in vivo through the CSE/H₂S pathway, demonstrating robust anti-inflammatory and antioxidant effects, further supporting its therapeutic potential for inflammatory joint disorders.

In TMJ-OA, the balance between ECM synthesis and degradation is disrupted, leading to cartilage degeneration (Cardoneanu et al. 2022). This imbalance is driven by the upregulation of matrix-degrading enzymes, such as MMPs and ADAMTS, alongside the downregulation of cartilage anabolic factors(Wang et al. 2015). IL-1β, a critical pro-inflammatory cytokine implicated in TMJ-OA, is commonly used to simulate the inflammatory microenvironment of TMJ-OA in vitro (Chen et al. 2024; Rajendiran et al. 2023). In our study, IL-1β markedly increased the expression of cartilage degradation factors, including MMP3, MMP9, MMP13, and COX2, while it suppressed the expression of cartilage anabolic factors, such as SOX-9 and aggrecan. SPRC effectively mitigated the inflammatory effects of COX2 and reduced ECM degradation in rPCCs. However, our data indicated that SPRC did not directly affect the expression of cartilage anabolic enzymes. We hypothesized that the therapeutic effects of SPRC in TMJ-OA are not entirely or directly attributable to its action on condylar chondrocytes.

There is intricate crosstalk between the synovium and cartilage in TMJ-OA (Wu et al. 2024). Previous studies have demonstrated that treating chondrocytes with macrophage-CM effectively simulates the TMJ microenvironment, with CM derived from M2 macrophages significantly promoting cartilage regeneration in TMJ-OA (Wu et al. 2023; Xia et al. 2024). In our co-culture experiments, CM from SPRC-treated macrophages increased chondrocyte metabolic activity, restored ECM integrity, and facilitated cartilage differentiation. These findings suggest that SPRC promotes a favorable microenvironment for cartilage repair. Notably, chondrocytes possess secretory functions, raising the question of whether CM from SPRC-treated chondrocytes could further enhance macrophage polarization—a hypothesis that warrants future investigation.

Despite the promising findings of this study, several limitations remain. First, although a 4-week intra-articular injection is a standard and effective treatment cycle for TMJ-OA in vivo experiments (Hei et al. 2024; Kaneko et al. 2000; Yang et al. 2024a, b), the long-term therapeutic effects of SPRC require further investigation. To address this, we plan to expand the study by incorporating additional animal models (including rats, sheep, and pigs) and evaluating disease progression at extended time points (e.g., 6 weeks, 8 weeks, and beyond) to assess the sustained impact of SPRC treatment better. Second, further research is necessary to elucidate the molecular mechanisms underlying SPRC’s therapeutic effects for clinical application. Future studies should clarify how SPRC regulates synovium-cartilage crosstalk, providing a stronger foundation for TMJ-OA treatment. These insights could facilitate the clinical translation of SPRC by supporting the development of combined therapeutic strategies, such as the co-administration of SPRC and HA, to enhance synergistic efficacy. Additionally, this research could advance phase I clinical trials, promoting the potential clinical adoption of SPRC for TMJ-OA treatment.

## Conclusions

This study underscores the therapeutic potential of SPRC in the treatment of TMJ-OA. SPRC effectively facilitated the repolarization of pro-inflammatory M1 macrophages to the anti-inflammatory M2 phenotype. The underlying mechanism appears to involve the modulation of the JAK2/STAT3 signaling pathway, leading to reduced synovial inflammation and cartilage degradation. Furthermore, SPRC protected against cartilage damage induced by macrophage-CM and IL-1β stimulation. These findings suggest that SPRC represents a promising therapeutic candidate for TMJ-OA, providing valuable insights into novel strategies targeting macrophage polarization and synovium-cartilage crosstalk.

## Electronic supplementary material

Below is the link to the electronic supplementary material.

**Supplementary Material 1:** Figure S1

**Supplementary Material 2:** Figure S2

**Supplementary Material 3:** Western Blot Raw Data

## Data Availability

No datasets were generated or analysed during the current study.

## Abbreviations

TMJ-OA: Temporomandibular joint osteoarthritis
TMD: Temporomandibular joint disorder
H_2_S: Hydrogen sulfide
ROS: Reactive oxygen species
NO: Nitric oxide
ROS: Reactive oxygen species
NO: Nitric oxide
CBS: Cystathionine β-synthase
CSE: Cystathionineγ-lyase
3-MST: 3-mercaptopyruvate sulfurtransferase
SPRC: S-propargyl-cysteine
OA: Osteoarthritis
HWT: Head withdrawal threshold
PFA: Paraformaldehyde
BV/TV: Total tissue volume
Tb.Sp: Trabecular separation
BMD: Bone mineral density
H&E: Hematoxylin and eosin
S&F: Safranin-O/fast green
LPS: Lipopolysaccharide
rPCCs: Rat primary condylar chondrocytes
cDNA: Complementary DNA
RT-qPCR: Realtime fluorescence quantitative PCR
CM: Conditioned medium
Hcy: Homocysteine
GSEA: Gene Set Enrichment Analysis
MMPs: Matrix metalloproteinases
ECM: Extracellular matrix
COX-2: Cyclooxygenase-2
