# Correction: S-propargyl-cysteine attenuates temporomandibular joint osteoarthritis by regulating macrophage polarization via Inhibition of JAK/STAT signaling

**DOI:** 10.1186/s10020-025-01214-5

**Published:** 2025-04-28

**Authors:** Wenyi Cai, Antong Wu, Zhongxiao Lin, Wei Cao, Janak L. Pathak, Richard T. Jaspers, Rui Li, Xin Li, Kaihan Zheng, Yufu Lin, Na Zhou, Xin Zhang, Yizhun Zhu, Qingbin Zhang

**Affiliations:** 1 Department of Temporomandibular Joint, School and Hospital of Stomatology, Guangdong Engineering Research Center of Oral Restoration and Reconstruction, Guangzhou Key Laboratory of Basic and Applied Research of Oral Regenerative Medicine, Guangzhou Medical University, 195 Dongfeng Road (West), Yuexiu District, Guangzhou, Guangdong 510140 China; 2 Laboratory for Myology, Department of Human Movement Sciences, Faculty of Behavioural and Movement Sciences, Vrije Universiteit Amsterdam, Amsterdam Movement Science, Amsterdam, The Netherlands; 3 School of Pharmacy, State Key Laboratory of Quality Research in Chinese Medicines and Laboratory of Drug Discovery from Natural Resources and Industrialization, Macau University of Science and Technology, Room 210, Block E, Avenida Wai Long, Taipa, Macau China; 4 Guangzhou Municipal and Guangdong Provincial Key Laboratory of Molecular Target & Clinical Pharmacology, The NMPA and State Key Laboratory of Respiratory Disease, School of Pharmaceutical Sciences, Guangzhou Medical University, Guangzhou, 511436 China

**Correction: Molecular Medicine (2025) 31:128**

10.1186/s10020-025-01186-6

In this article, Fig. 6 appeared incorrectly and have now been corrected in the original publication. For completeness and transparency, both correct and incorrect versions are displayed below.

Incorrect Fig. 6.

**Fig. 6:**
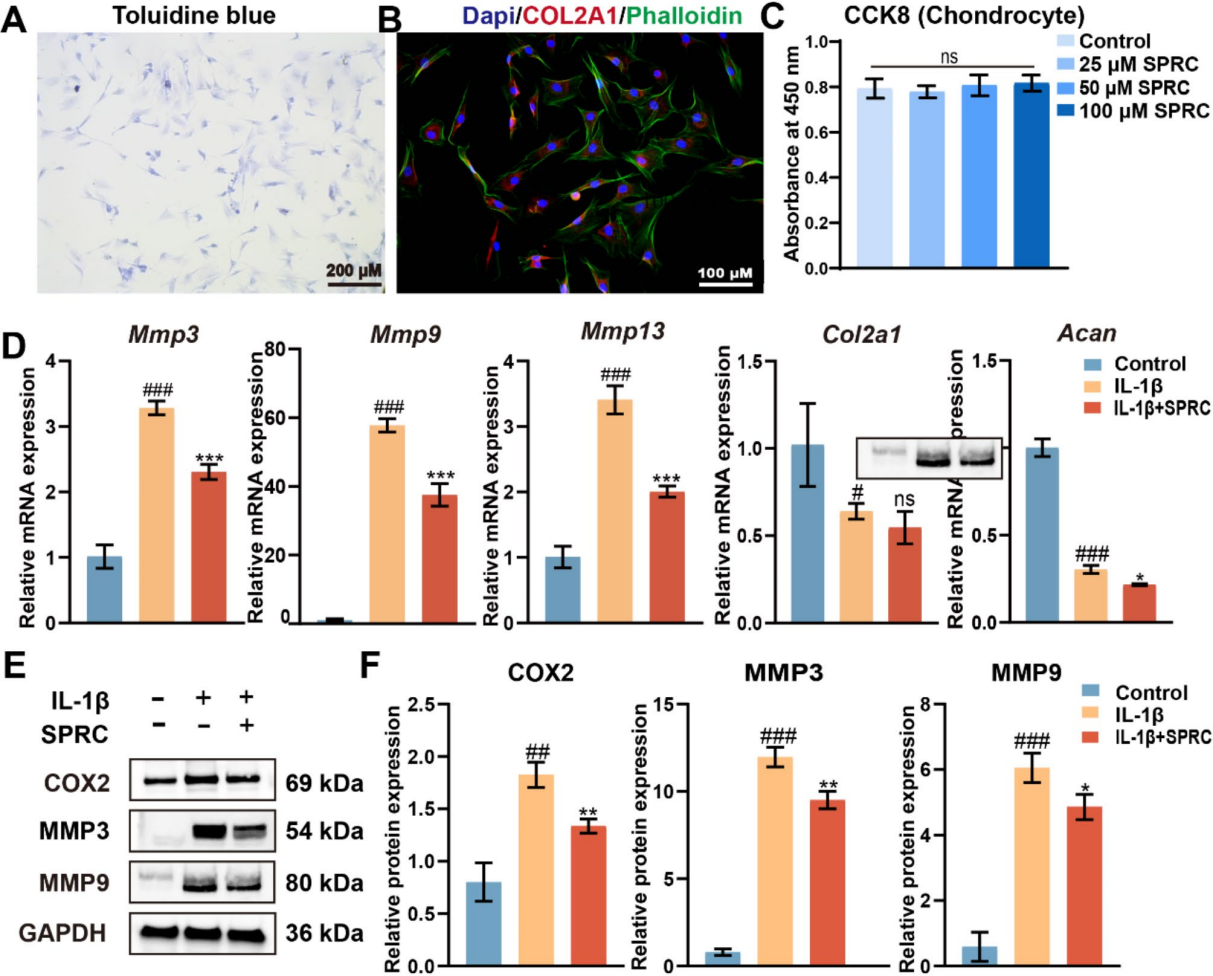
SPRC reduced condylar chondrocytes ECM catabolism in vitro. (**A**) Representative images of condylar chondrocytes stained with toluidine blue to assess extracellular matrix (ECM) integrity. (**B**) Immunofluorescence staining of chondrocytes showing DAPI-stained nuclei (blue), phalloidin-stained F-actin (green), and COL2A1 (red). (**C**) Cell viability of rat primary condylar chondrocytes (rPCCs) treated with SPRC (25, 50, or 100 μM) for 24 h, as evaluated by the CCK8 assay. (**D**) RT-qPCR analysis of matrix degradation-related genes (Mmp3, Mmp9, and Mmp13) and matrix synthesis-related genes (Col2a1 and Acan). (**E**-**F**) Western blot analysis of COX2, MMP3, and MMP9 protein expression levels. The data were analyzed via one-way ANOVA (n ≥ 3). Statistical significance is denoted as follows: #p<0.05, ##p < 0.01, and ###p < 0.001 compared with the control group; *p < 0.05, **p < 0.01, and ***p < 0.001 compared with the IL-1β group. “ns” indicates no significance

Correct Fig. 6.

**Fig. 6:**
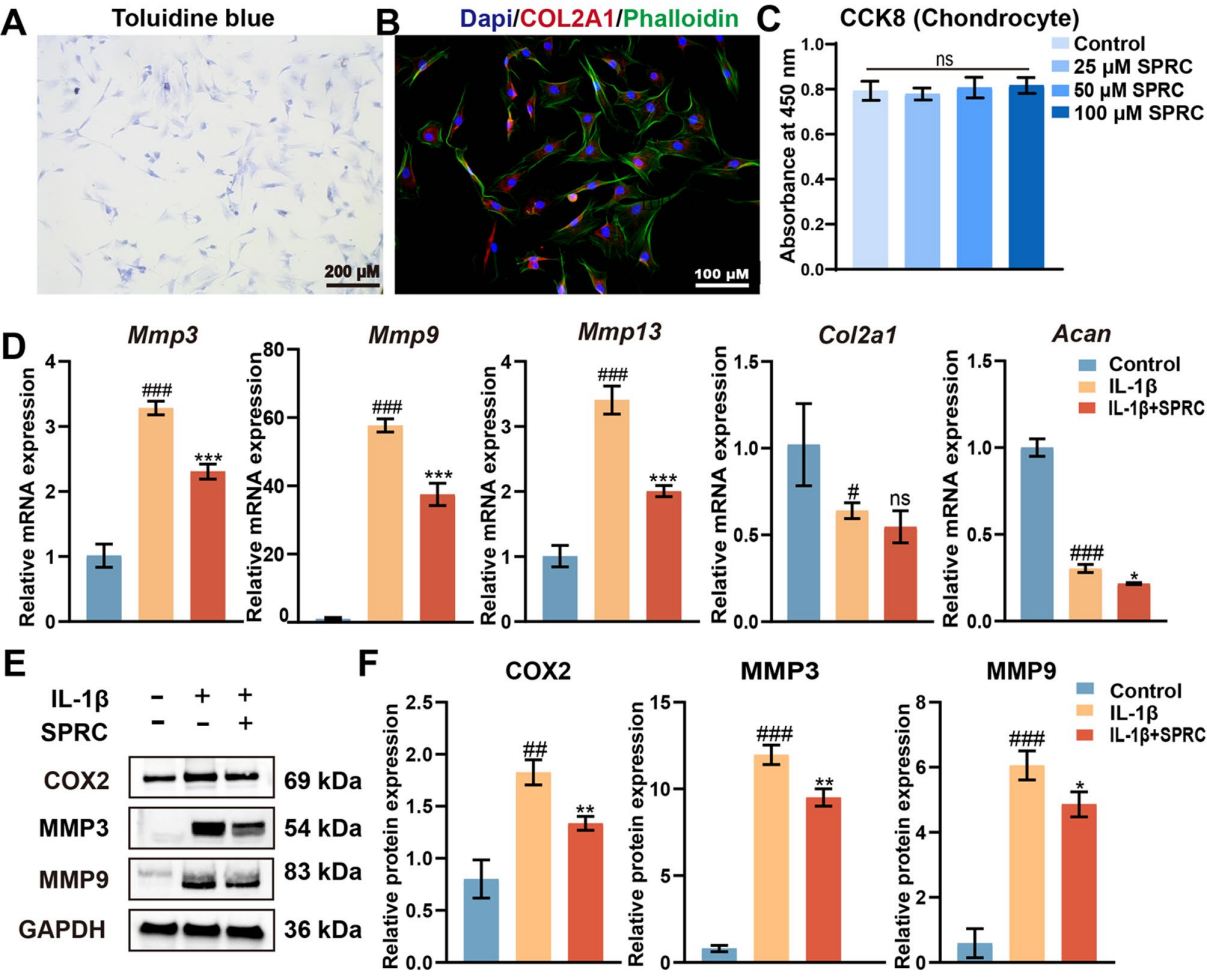
SPRC reduced condylar chondrocytes ECM catabolism in vitro. (**A**) Representative images of condylar chondrocytes stained with toluidine blue to assess extracellular matrix (ECM) integrity. (**B**) Immunofluorescence staining of chondrocytes showing DAPI-stained nuclei (blue), phalloidin-stained F-actin (green), and COL2A1 (red). (**C**) Cell viability of rat primary condylar chondrocytes (rPCCs) treated with SPRC (25, 50, or 100 μM) for 24 h, as evaluated by the CCK8 assay. (**D**) RT-qPCR analysis of matrix degradation-related genes (Mmp3, Mmp9, and Mmp13) and matrix synthesis-related genes (Col2a1 and Acan). (**E**-**F**) Western blot analysis of COX2, MMP3, and MMP9 protein expression levels. The data were analyzed via one-way ANOVA (n ≥ 3). Statistical significance is denoted as follows: #p<0.05, ##p < 0.01, and ###p < 0.001 compared with the control group; *p < 0.05, **p < 0.01, and ***p < 0.001 compared with the IL-1β group. “ns” indicates no significance

The original article has been corrected.
