# Supplementary figures and images for: S-propargyl-cysteine attenuates temporomandibular joint osteoarthritis by regulating macrophage polarization via Inhibition of JAK/STAT signaling
Source: Mol Med. 2025 Apr 7;31:128. doi: 10.1186/s10020-025-01186-6 (PMC11974036; doi:10.1186/s10020-025-01186-6)

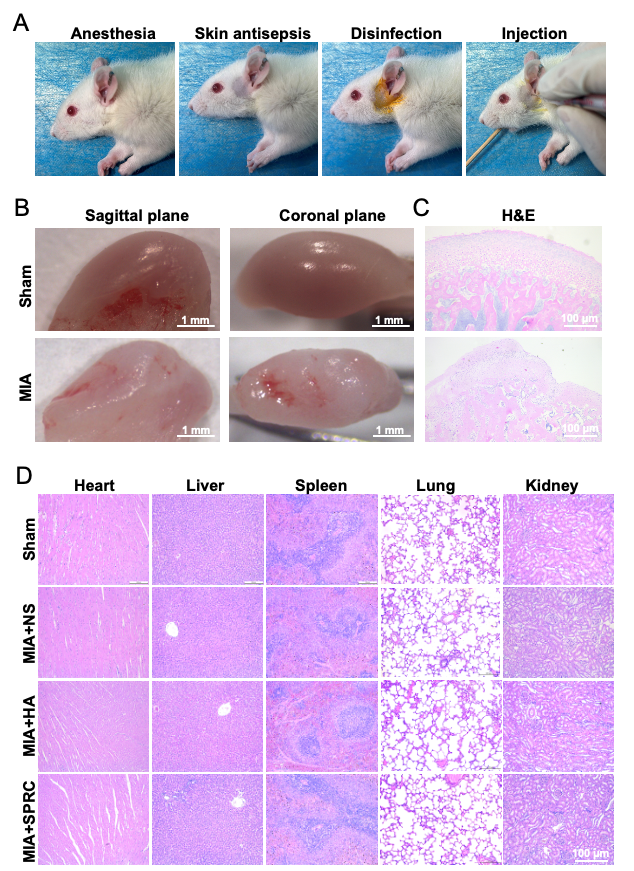

Supplement: Supplementary file 1 — Supplementary Material 1: Figure S1 [file 10020_2025_1186_MOESM1_ESM.tiff]

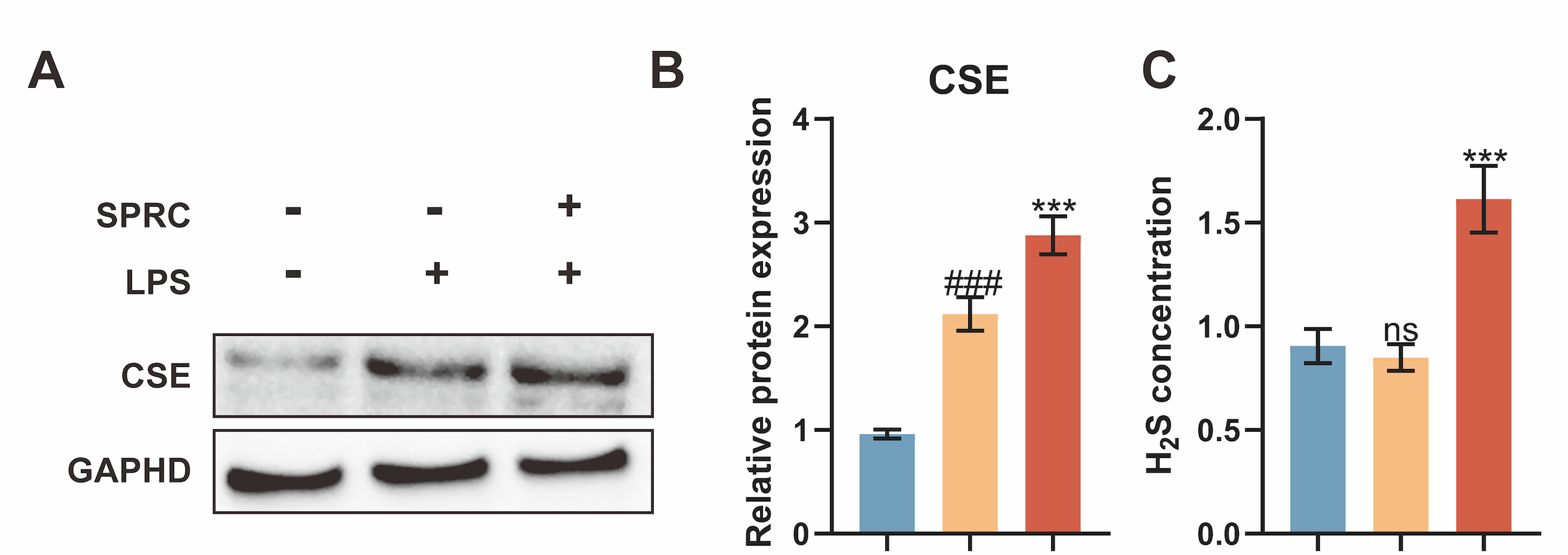

Supplement: Supplementary file 2 — Supplementary Material 2: Figure S2 [file 10020_2025_1186_MOESM2_ESM.tif]
