# Supplementary material for: S-propargyl-cysteine attenuates temporomandibular joint osteoarthritis by regulating macrophage polarization via Inhibition of JAK/STAT signaling
Source: Mol Med. 2025 Apr 7;31:128. doi: 10.1186/s10020-025-01186-6 (PMC11974036; doi:10.1186/s10020-025-01186-6)

Figure 4 E

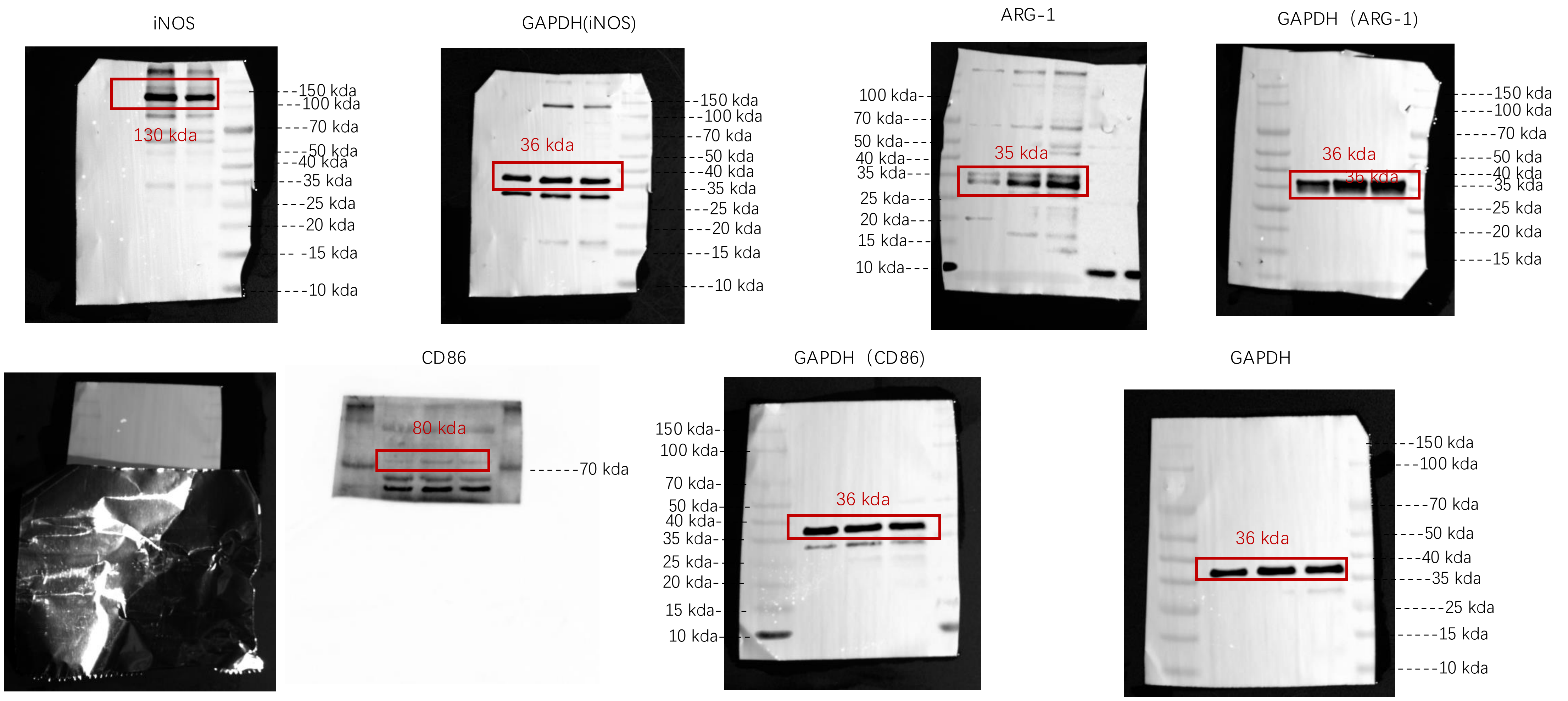
iNOS
130 kda
--150 kda
---100 kda
----70 kda
----50 kda
----40 kda
---35 kda
---25 kda
---20 kda
-- -15 kda
---10 kda
GAPDH(iNOS)
36 kda
--150 kda
--100 kda
--70 kda
--50 kda
--40 kda
--35 kda
--25 kda
--20 kda
---15 kda
---10 kda
ARG-1
35 kda
100 kda-
70 kda---
50 kda---
40 kda---
35 kda---
25 kda---
20 kda---
15 kda---
10 kda---
GAPDH（ARG-1)
36 kda
----150 kda
----100 kda
-----70 kda
----50 kda
----40 kda
----35 kda
----25 kda
----20 kda
----15 kda
CD86
80 kda
------70 kda
GAPDH（CD86)
36 kda
150 kda-
100 kda-
70 kda-
50 kda-
40 kda-
35 kda-
25 kda-
20 kda-
15 kda-
10 kda-
GAPDH
36 kda
--150 kda
---100 kda
----70 kda
---50 kda
---40 kda
----35 kda
------25 kda
-----15 kda
-----10 kda

Figure 5 B

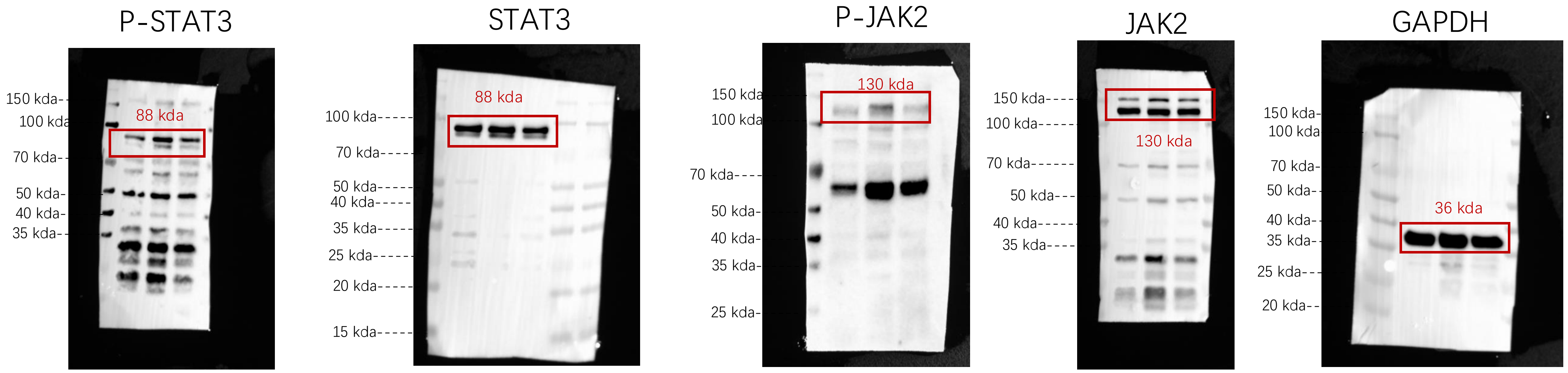

Figure 5 E

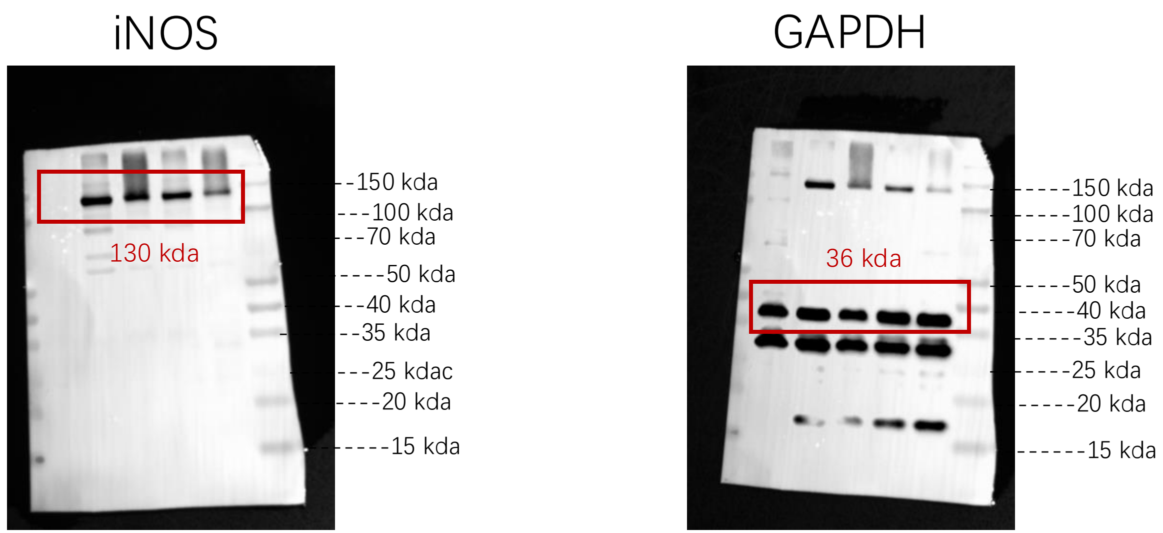

Figure 6 E

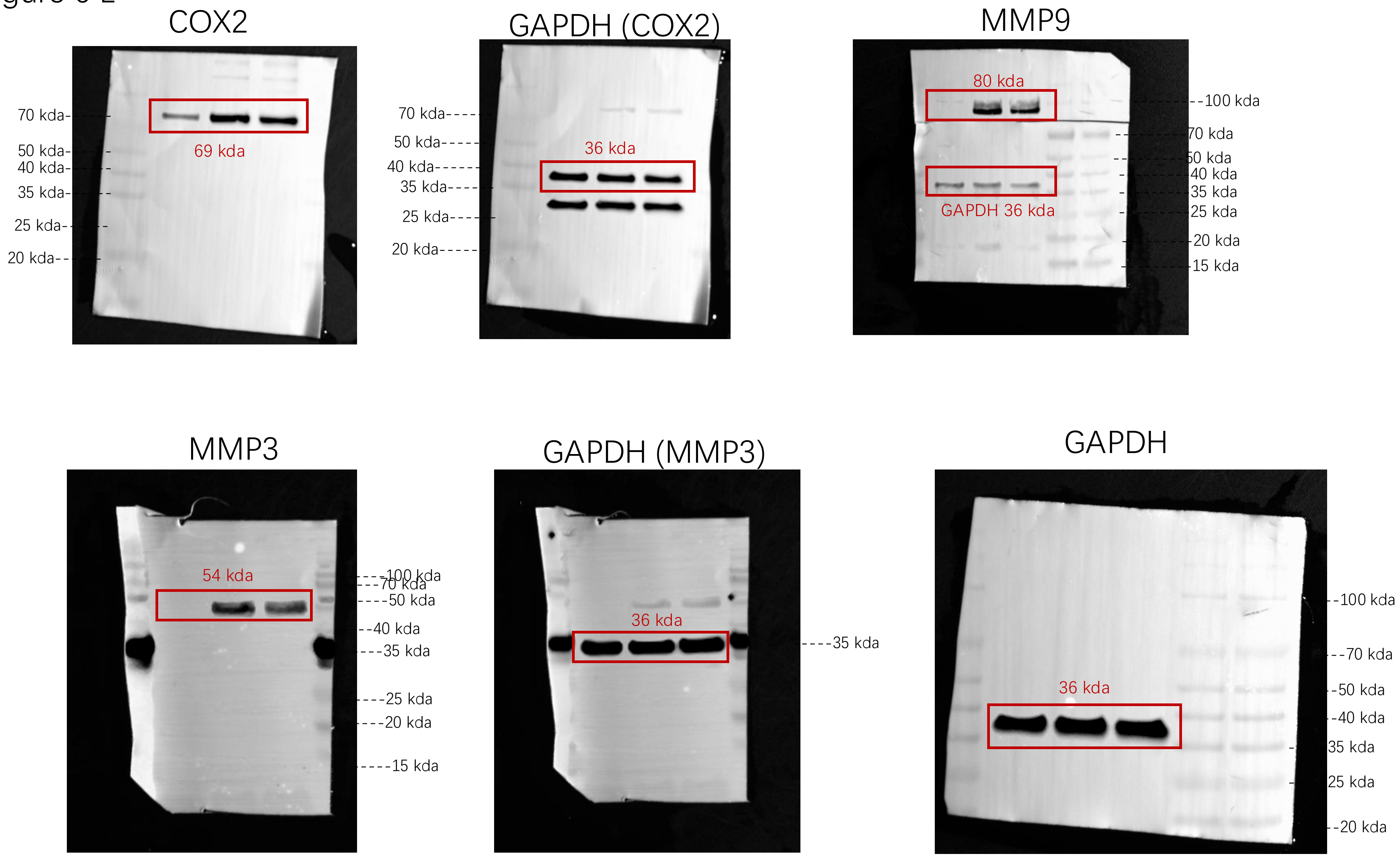

COX2
70 kda
50 kda
40 kda
35 kda
25 kda
20 kda
69 kda
GAPDH (COX2)
70 kda
50 kda
40 kda
35 kda
25 kda
20 kda
36 kda
MMP9
80 kda
100 kda
70 kda
50 kda
40 kda
35 kda
25 kda
20 kda
15 kda
GAPDH 36 kda
MMP3
54 kda
100 kda
70 kda
50 kda
40 kda
35 kda
25 kda
20 kda
15 kda
GAPDH (MMP3)
36 kda
35 kda
GAPDH
36 kda
100 kda
70 kda
50 kda
40 kda
35 kda
25 kda
20 kda

Figure 7 D

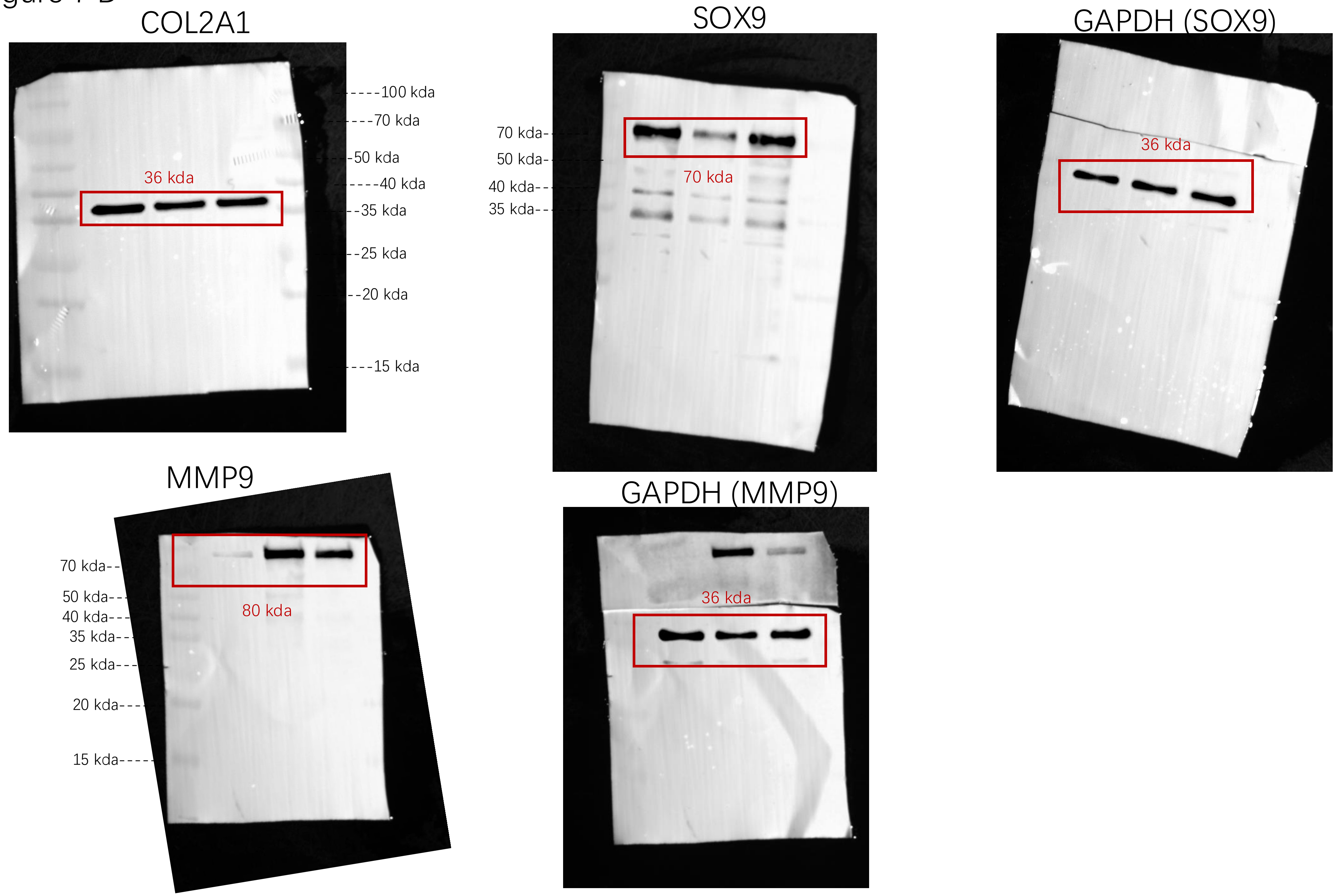
COL2A1
36 kda
100 kda
70 kda
50 kda
40 kda
35 kda
25 kda
20 kda
15 kda
SOX9
70 kda
70 kda
50 kda
40 kda
35 kda
GAPDH (SOX9)
36 kda
MMP9
80 kda
70 kda
50 kda
40 kda
35 kda
25 kda
20 kda
15 kda
GAPDH (MMP9)
36 kda

Supplymentary Figure 2

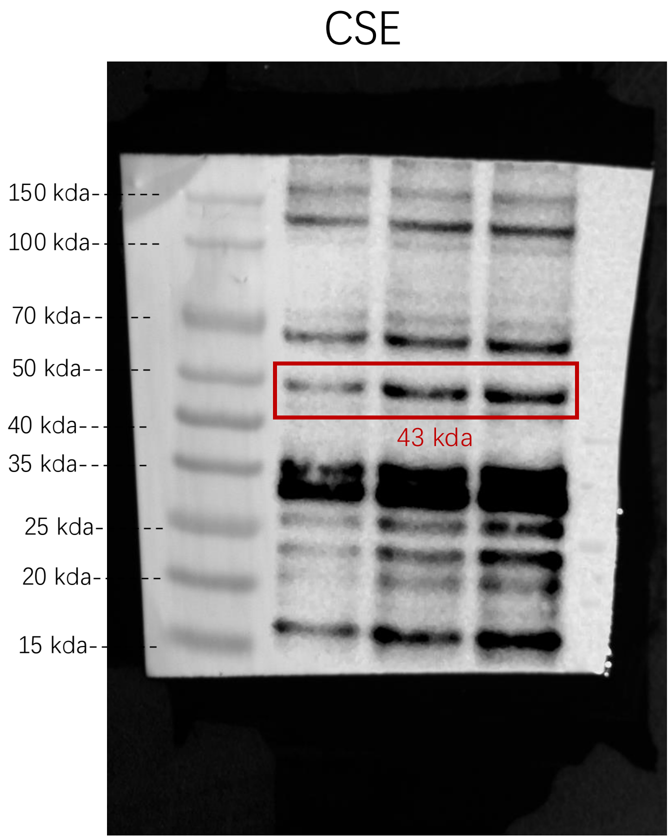

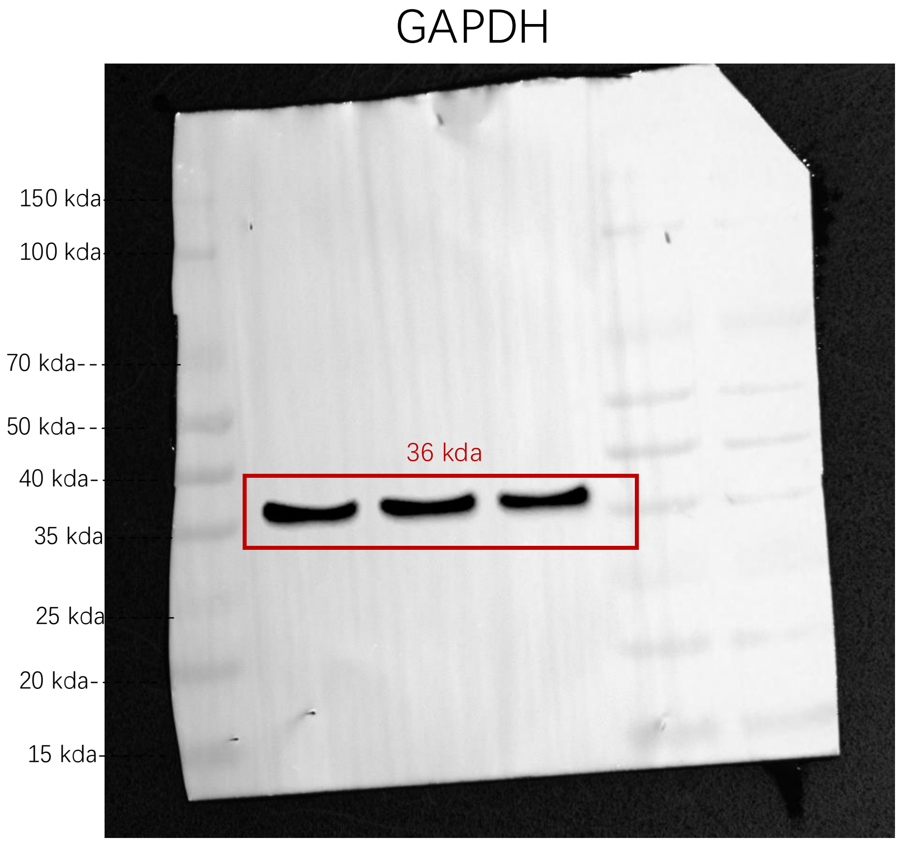

Supplement: Supplementary file 3 — Supplementary Material 3: Western Blot Raw Data [file 10020_2025_1186_MOESM3_ESM.pdf]
